# ARCHERY: a prospective observational study of artificial intelligence-based radiotherapy treatment planning for cervical, head and neck and prostate cancer – study protocol

**DOI:** 10.1136/bmjopen-2023-077253

**Published:** 2023-12-07

**Authors:** Ajay Aggarwal, Laurence Edward Court, Peter Hoskin, Isabella Jacques, Mariana Kroiss, Sarbani Laskar, Yolande Lievens, Indranil Mallick, Rozita Abdul Malik, Elizabeth Miles, Issa Mohamad, Claire Murphy, Matthew Nankivell, Jeannette Parkes, Mahesh Parmar, Carol Roach, Hannah Simonds, Julie Torode, Barbara Vanderstraeten, Ruth Langley

**Affiliations:** 1 Institute of Clinical Trials and Methodology - MRC CTU at UCL, University College London, London, UK; 2 Faculty of Public Health and Policy, London School of Hygiene and Tropical Medicine, London, UK; 3 MD Anderson Cancer Center, University of Texas, Houston, Texas, USA; 4 Department of Oncology, The Christie NHS Foundation Trust, Manchester, UK; 5 National Radiotherapy Trials Quality Assurance Group, Mount Vernon Hospital, Northwood, UK; 6 Department of Radiation Oncology, Tata Memorial Hospital, Mumbai, Maharashtra, India; 7 Ghent University Hospital, Gent, Belgium; 8 Department of Radiation Oncology, Tata Memorial Center, Kolkata, West Bengal, India; 9 University of Malaya, Kuala Lumpur, Wilayah Persekutuan, Malaysia; 10 King Hussein Cancer Center, Amman, Jordan; 11 University of Cape Town, Rondebosch, South Africa; 12 Stellenbosch University, Stellenbosch, Western Cape, South Africa; 13 King’s College London, London, UK

**Keywords:** RADIOTHERAPY, ONCOLOGY, Adult oncology

## Abstract

**Introduction:**

Fifty per cent of patients with cancer require radiotherapy during their disease course, however, only 10%–40% of patients in low-income and middle-income countries (LMICs) have access to it. A shortfall in specialised workforce has been identified as the most significant barrier to expanding radiotherapy capacity. Artificial intelligence (AI)-based software has been developed to automate both the delineation of anatomical target structures and the definition of the position, size and shape of the radiation beams. Proposed advantages include improved treatment accuracy, as well as a reduction in the time (from weeks to minutes) and human resources needed to deliver radiotherapy.

**Methods:**

ARCHERY is a non-randomised prospective study to evaluate the quality and economic impact of AI-based automated radiotherapy treatment planning for cervical, head and neck, and prostate cancers, which are endemic in LMICs, and for which radiotherapy is the primary curative treatment modality. The sample size of 990 patients (330 for each cancer type) has been calculated based on an estimated 95% treatment plan acceptability rate. Time and cost savings will be analysed as secondary outcome measures using the time-driven activity-based costing model. The 48-month study will take place in six public sector cancer hospitals in India (n=2), Jordan (n=1), Malaysia (n=1) and South Africa (n=2) to support implementation of the software in LMICs.

**Ethics and dissemination:**

The study has received ethical approval from University College London (UCL) and each of the six study sites. If the study objectives are met, the AI-based software will be offered as a not-for-profit web service to public sector state hospitals in LMICs to support expansion of high quality radiotherapy capacity, improving access to and affordability of this key modality of cancer cure and control. Public and policy engagement plans will involve patients as key partners.

**STRENGTHS AND LIMITATIONS OF THIS STUDY**

- Study evaluates artificial intelligence (AI) in a broad range of low-income and middle-income countries future roll-out and scale up.
- Prospective evaluation of all eligible participants per study site to assess factors influencing selection into study.
- Participating centres need intensity modulated radiotherapy availability with routine use for prostate, cervix and head and neck cancer.
- Automated contours and plan will be assessed by double-blind peer review according to international quality standards but not directly compared with the manual plan.
- Comparison of time savings between automated and manual pathways and the budget impact of integrating AI routinely into the clinical workflow.

## Introduction

By 2030, new cancer cases worldwide are projected to rise to 21.3 million annually, of which approximately 70% will be from low-income and middle-income countries (LMICs). Radiotherapy (RT) is a core modality of cancer control and cure for several types of cancer that are common in these settings such as cancers of the cervix, prostate, and head and neck, with 50% of such patients requiring RT during their disease course.^1 2^ However, only 10% of patients in low-income, and 40% of patients in middle-income countries, have access to RT.^3^ Limited resources have contributed to long waiting times for treatment resulting in cancer progression, increased morbidity and inferior survival outcomes, as well as high rates of impoverishing health expenditures.^4^

The WHO has set a target, which states that RT is an essential medical device and should be available to 80% of the world’s population by 2025.^5^ It also recognises that a critical shortfall in the specialised workforce required to deliver RT is the most significant barrier to access.^1^ The Global Taskforce on RT for Cancer Control estimated that by 2035, an additional 30 000 radiation oncologists (RO), 22 000 medical physicists (MPs) and 80 000 treatment radiographers (RTT) would be required to meet demand.^1^

These workforce shortfalls are coupled with a complex RT treatment workflow, requiring several labour-intensive processes from highly trained people including ROs, MPs, dosimetrists (DOs) and RTTs.^6^ The process starts with a dedicated RT CT planning scan, followed by the clinician manually segmenting the (a) tumour (gross tumour volume (GTV)) and (b) areas that are at risk of tumour spread (clinical target volumes (CTVs), eg, regional lymph nodes); organs at risk (OARs) of radiation damage such as the spinal cord, salivary glands, bladder and rectum. This can take 2–3 hours per patient even in the hands of an experienced practitioner. Next, MPs or DOs define the position, size and shape of the radiation beams to ensure adequate coverage of target structures with radiation at the correct dose which can take up to a further half a day.

This pretreatment RT workflow can typically take up to 4 weeks in high-income countries and as long as 12 weeks in LMICs due to patient demand and workforce shortages. In addition, errors and delays can accumulate at each stage of RT planning which can negatively impact the quality of treatment. This includes interclinician variability in target volume delineation which can have a detrimental impact on patient outcomes.^7^

Workforce and skills shortages and lack of financing means countries have been unable to transition from two-dimensional X-ray-based planning to CT-based methods (eg, three-dimensional conformal and intensity modulated RT (IMRT)), which are associated with better outcomes.^8^

The growing demand for RT means that a scalable solution is required to address these challenges. In this regard, a recent WHO report highlighted the potential of digital technologies such as artificial intelligence (AI) to contribute to advancing universal health coverage achieving the Sustainable Development Goals such as ensuring equity in access to treatments and their affordability.^9^

The radiotherapy planning assistant (RPA) is an AI-based software that has been developed to automate two key components of the RT planning pathway:

1. Contouring of anatomical areas that are at risk of tumour spread (CTVs) and at risk of radiation damage (OARs).
2. Definition of the position, size and shape of the radiation beams to the target organs.

The AI-based contouring models that are included in the RPA were developed by the University of Texas MD Anderson Cancer Centre.^10 11^ User interfaces and AI-based planning for conformal RT treatments were also developed,^12–14^ as were in-built quality assurance tools,^15 16^ and the training and testing of the knowledge-based planning component of the automated planning software which is a function of the Eclipse treatment planning system (Varian Medical Systems). To the best of our knowledge, the RPA is the only application ready for clinical use in head and neck, cervical and prostate cancer which can both contour CTVs and OARs and produce an optimised treatment plan.

The ARCHERY study is a prospective international multicentre study in India, Jordan, Malaysia and South Africa to evaluate the quality, time, resource use and cost associated with using the RPA for the treatment of primary cervical, head and neck, and prostate cancers. The first two cancers represent sites which are leading causes of cancer death in LMICs.^17 18^ In addition, provision of effective, equitable and affordable RT is vital in order to achieve the WHO’s target of eliminating cervical cancer as a public health problem.^19^ Prostate cancer represents a very common cancer where RT plays an important curative role.^17^

We will assess whether the RPA can automate target volume contouring including OARs and produce RT plans that are able to meet international standards for treatment quality. This would also establish whether AI computer-based software can be routinely integrated into the workflow of diverse high volume LMIC centres which vary in their technical and workforce infrastructure to support the AI integration. The study aims to evaluate the extent to which routine use of the RPA will support better workforce allocation and reduce the costs of RT treatment delivery. LMIC centres have been chosen in this evaluation as they have the greatest need at present; however, the study and its goals are also relevant in the HIC setting and therefore the appraisal of the RPA will use current international quality assurance standards rather than direct comparison of outcomes achieved within individual RT centres.^20^

## Methods and analysis

### Patient inclusion criteria

1. Patients with:
  - Histologically confirmed head and neck cancers of the oropharynx, larynx, hypopharynx and nasopharynx (American Joint Committee on Cancer (AJCC) stages I–IVB) that has given consent for radical RT. Induction chemotherapy prior to RT acceptable.
    OR
  - Histologically confirmed primary cervical cancer (International Federation of Gynaecology and Obstetrics/AJCC stages IB–IIIC1) that have given consent for radical RT. Induction chemotherapy prior to RT or concurrent chemoradiotherapy acceptable.
    OR
  - Histologically confirmed primary prostate cancer (AJCC stages I–IIIC) that have given consent for radical RT.
2. Provide signed informed consent to participate in the study.
3. Aged ≥18 years.

### Patient exclusion criteria

1. Patients requiring RT after curative surgery or surgery that is intended to remove as much of the tumour as possible.
2. Patients receiving palliative RT.

Consecutive patients who are eligible will be approached and screening data collected to establish the proportion of eligible patients that consent to participate. Participants are only eligible for the study if they have already provided consent to receive radical RT and participation in ARCHERY will not change how the patient is treated.

### Site exclusion criteria

1. Centre using exclusively cobalt-based RT for treatment.
2. Centres not using standardised international guidelines/protocols for target volume contouring and dosimetric radiation constraints.

### Study conduct

The AI-based RT planning tool (the RPA) will be applied to the CT scan acquired as part of a participants planned RT treatment process.

The RPA will be used to automate:

1. CTV and OAR contouring.
2. Treatment planning.

Randomisation is not required as the initial CT scan of the participant, which is used as the template for contouring and planning, will be used for both the standard and automated approaches. Patients will be treated on the standard plan and will not be affected by the study. Local centres will remain blinded to the results of the automated plans.

### CTVs and OAR contouring

Contours will be applied manually to the primary tumour GTV (eg, for head and neck) by the treating clinician.

**Figure 1:**
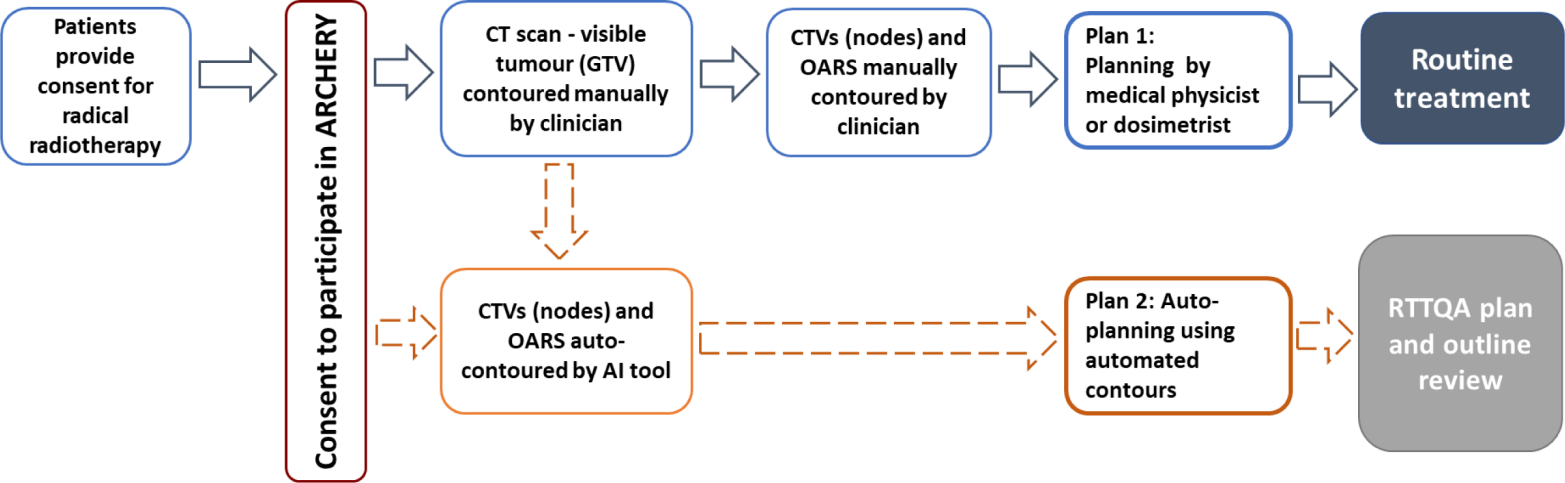
Study workflow. CTV, clinical target volume; GTV, gross tumour volume; OAR, organs at risk; RTTQA, RT Trials Quality Assurance.

This CT scan with the contoured GTV will be used for the manual and the automated study pathway (figure 1) to create plans 1 and 2. The contouring of CTVs and OARs will be performed by the clinician (blue boxes in figure 1) or the automated by AI using the RPA (orange boxes in figure 1).

For the prostate cancer pathway, there will be no GTV to be outlined but a CTV (prostate gland±seminal vesicles) as per current international guidance which will be outlined by the clinician or the RPA.

### Treatment planning

Two plans will be produced for evaluation. Plan 1: Manual contours will undergo planning using standard clinical protocols by a DO or physicist at the local site, and this will be used to treat the patient (blue box in figure 1). Plan 2: The automated contours will undergo autoplanning and this will undergo clinical evaluation (orange box in figure 1).

### Quality assessment protocol

The final RT plans will be exported as DICOM (Digital Imaging and Communication in Medicine) files, to the UK RT Trials Quality Assurance (RTTQA) group who will coordinate the assessment of contouring (CTVs and OARs) and planning (dose distribution) produced by the RPA. An international panel all with greater than 10 years of experience as an RO will undertake the evaluation of the treatment contours using a protocol based on international standards for target volume definition and dosimetry.

### Procedures for assessing efficacy

The primary outcome measure is overall treatment plan acceptability, a composite outcome including both the assessment of the delineated CTVs and OARs with reference to internationally accepted contouring guidelines and the adherence to predefined dosimetric constraints (table 1). We will not collect patient outcomes following treatment as important clinical differences in terms of toxicity and tumour control are not expected if these validated criteria are met and patients will not be treated on the automated plans. In addition, variation in the accuracy of patients set up across the centres for treatment may result in important differences in patient outcomes unrelated to the quality of the manual or automated contours.^21 22^

**Table 1:** Assessment procedures for each treatment plan based on a composite score

Contouring	Dosimetry	Overall assessment
Score 3 or 4	Score 3 or 4	Acceptable
Score 3 or 4	Score 1 or 2	Unacceptable
Score 1 or 2	Score 3 or 4	Unacceptable
Score 1 or 2	Score 1 or 2	Unacceptable

The automated plan (plan 2) will be analysed against the expected plan acceptability rate from the literature which estimates that 95% of RT plans will be acceptable (scores 3 and 4 according to study scoring criteria) for treatment without the requirement for major edits (scores of 1 or 2).^7 23^ Major edits (as defined using the Global Quality Assurance of Radiation Therapy Clinical Trials criteria) are edits that if not undertaken would affect the likelihood of cure, locoregional control and could result in additional toxicity.^24^

The assessment of the contours and dosimetry from the automated plans will be coordinated by the RTTQA group and use international standards and procedures for RT planning quality assurance which have been agreed by the Global Quality Assurance of Radiation Therapy Clinical Trials Harmonisation Group (GHG) https://rtqaharmonization.org.

The RTTQA group will develop procedures and guidelines for OAR and CTV contour assessment in line with international standards for normal tissue and nodal CTVs for cervical, head and neck and prostate cancer^25–30^ that will be used by the peer reviewers in their assessments.

A scoring framework developed with reference to GHG protocol variation definitions will be provided for assessment of contouring with examples given of contours considered clinically not acceptable. The Likert scale below (with potential scope for minor changes) will be used for the grading of contouring:

- Score 4—Acceptable—per protocol: No edits required. The current contours are clinically acceptable and could be used for treatment without change.
- Score 3—Acceptable variation: Minor edits, not expected to have a major clinical impact on treatment outcome. Edits to the current contours should be considered.
- Score 2—Unacceptable variation: Major edits, may affect the likelihood of cure, locoregional control and potentially cause additional toxicity. The current contours are clinically unacceptable.
- Score 1—Unacceptable variation with treatment modification: Major edits, however, these variations could not have been reasonably avoided due to clinical necessity as perceived by the treating physician.

Two reviewers will independently review each set of contours. If there is any discordance between the two reviewers, that is, an unacceptable score (1 or 2) by one reviewer and acceptable score (3 or 4) by another, a third reviewer will be consulted, and acceptability will be defined based on a consensus majority rule (2:1).

The dosimetric assessment of the plans will be automated using the autoextracted dose volume histogram (DVH) constraints. The framework for assessment will be defined for each tumour cohort with reference to GHG criteria.^31^ If any of the DVH constraints are not met, the plan would be assessed independently by two peer reviewers to evaluate whether there is any mitigation for the dosimetric constraints not being met (eg, extensive tumour), otherwise the plan will be graded as clinically unacceptable.

Dosimetric constraints will be based on contemporary RT IMRT RCT protocols for cervical, head and neck, and prostate cancer^27 32 33^ but will also require an understanding of differences in protocols used across different regions. The Likert scale below will be used for grading of DVHs:

- Score 4—Acceptable—per protocol: The radiation therapy treatment was planned according to protocol specifications. Mandatory and optimal volume planning constraints are met.
- Score 3—Acceptable variation: The radiation therapy treatment was not planned according to all the protocol specifications, but not expected to have a major clinical impact on treatment outcome. Mandatory volume planning constraints are met but not all the optimal volume planning constraints are achieved.
- Score 2—Unacceptable variation: The radiation therapy treatment was not planned according to protocol specifications and may affect the likelihood of cure, locoregional control and potentially cause additional toxicity.
- Score 1—Unacceptable variation with treatment modification: The radiation therapy treatment was not planned according to protocol specifications and may affect the likelihood of cure, locoregional control and potentially cause additional toxicity. However, these variations could not have been reasonably avoided due to clinical necessity as perceived by the treating physician.

### Quantifying and modelling the time cost savings from using the RPA by using the ESTRO approved time-driven activity-based costing model

To determine the time savings from the automated approaches compared with the manual approach, time-and-motion studies will be undertaken to populate the ESTRO time-driven activity-based costing model.^6^ This is considered the most contemporary and robust mechanism for radiation therapy costing and will be used to estimate the cost of delivering RT treatment for each tumour type in each centre, in addition to computing the actual personnel and equipment resource utilisation.^6^

The model requires time estimates for all RT processes within the typical RT care pathway including time for contouring anatomy including edits, plan production, peer review, QA checks, plan acceptance and treatment delivery.^34^ All times will be stratified according to the specialist personnel involved and undertaken for each of the two study planning pathways outlined in figure 1, that is, the (1) standard manual pathway and (2) fully automated pathway.

Allied to this will be the collection of detailed information at the centre level of the available resources and their associated costs. This includes:

- Indirect resources: resources (and their costs) that cannot be directly assigned to a single treatment, that is, personnel, equipment and related infrastructure and maintenance including overheads.
- Direct material costs related to a particular treatment course (masks, fiducials, contrast material).
- Information on the number of RT courses delivered in the centre (by tumour type, treatment intent, fractionation, complexity).

The outputs from the workflow timings study and data collection on the resources available and courses delivered at each individual centre will be key inputs into the model to estimate differences in costs and resource needs for cervical cancer, head and neck cancer and prostate cancer treatment courses using the two approaches (fully automated vs standard).

Using this information, we will be able to assess the impact on resource needs and associated costs (in particular for specialist personnel, eg, ROs from scaling up automation to other cancer types, and undertake cost-minimisation and budget impact analyses).^35^ The costing and cost-saving calculations will be performed at the level of each country and centre. The budget impact analysis will inform the affordability of the intervention to the health system, in terms of the feasibility of expanding the number of patients that can be treated with RT.^36^

### Sample size

The sample size for each tumour population has been calculated based on an overall 95% plan acceptability rate, with a CI which is no wider than±5% in absolute terms around this figure This rate is considered necessary to support implementation of the software as the clinical community would be unlikely to accept a lower plan acceptability rate of, for example, 80%.

To achieve at least 90% power that the lower confidence limit for the acceptability plan will be greater than 90%, assuming alpha=0.05 (or one-sided 0.025) and a binomial distribution, the required sample size is approximately 330 for each tumour population.^37^

### Statistical analysis plan

We will fit a logistic regression model to the binary outcome of plan acceptability adjusted by the centre with cluster robust SEs. We calculate the overall marginal proportion estimate balanced for the centre, to calculate the robust CI that maintains the family-wise error rate at 0.05. An additional analysis will investigate the inter-rater agreement for the plan. For each cancer, this will be assessed by calculating the kappa statistic between the first two expert raters assessing plans, so excluding the conditional third rating.

### Completion of participant follow-up

Participants have no further follow-up after their RT CT planning scan. Clinical management is as per the centre’s standard of care.

### Patient and public involvement

Patient partners have directly informed the research question, and cancer types chosen. The need for inclusion of AI and this study across LMIC partners has been defined through three international global RT workshops between 2017 and 2019, attended by the chief investigator (AA) at The European Organization for Nuclear Research, and in the UK and Botswana. As well as patient partners, attendees included clinical, academic and policy stakeholders from LMICs, including Ghana, Botswana, South Africa, Nigeria, Zambia and Jordan

With respect to study design, patient partners have highlighted that patient follow-up in LMICs can be challenging, with very low rates of follow-up following treatment. This has, therefore, informed the primary outcome measure of ‘RT plan acceptability’ as a measure of treatment quality without the necessity for ongoing follow-up. Patients will be treated using the current standard of care to avoid any delays, with the appraisal of automated plans occurring offline but using the RT CT planning scan that is acquired as part of the standard treatment process

Patients will be involved in the dissemination strategy. The patient-involvement coordinator (JT) will work with community engagement and involvement partners in each of the participating countries to develop a programme for dissemination of results.

### Ethics

The protocol has received ethical approval from University College London (UCL) Research Ethics Committee and each of the six recruiting sites’ Institutional Review Board (Institutional Ethics Committee, Tata Memorial Hospital; Institutional Ethics Committee, Tata Medical Centre; University of Malaya Medical Research Ethics Committee; King Hussein Cancer Center Institutional Review Board; Stellenbosch University Health Research Ethics Committee; University of Cape Town Health Research Ethics Committee). International sites will comply with the principles of GCP as laid down by the ICH topic E6 (R2) and other applicable national regulations.

Although there will be no clinical impact of the intervention or risk to the patient as they will continue to be treated according to the standard treatment pathway and not receive any additional imaging intervention or treatment, protections have been put in place for the use of pseudonymised CT scans and treatment planning details stored as DICOM files. Patients will be enrolled with written informed consent.

## Dissemination

If the study objectives are met, the AI-based software will be offered as a not-for-profit web service to public sector state hospitals in LMICs. We anticipate this will support rapid adoption into daily practice and facilitate expansion of high-quality RT in these countries. This, in turn, will improve access, and affordability of this critical treatment option, and therefore, also improve survival and the quality of life of patients with cancer globally.

## Supplementary Material

Reviewer comments

Author's manuscript
