# Supplementary material for: ARCHERY: a prospective observational study of artificial intelligence-based radiotherapy treatment planning for cervical, head and neck and prostate cancer – study protocol
Source: BMJ Open. 2023 Dec 7;13(12):e077253. doi: 10.1136/bmjopen-2023-077253 (PMC10711912; doi:10.1136/bmjopen-2023-077253)
Supplement: Author's manuscript [file bmjopen-2023-077253.draft_revisions.pdf]

BMJ Open

BMJ Open is committed to open peer review. As part of this commitment we make the peer review history of every article we publish publicly available.

When an article is published we post the peer reviewers' comments and the authors' responses online. We also post the versions of the paper that were used during peer review. These are the versions that the peer review comments apply to.

The versions of the paper that follow are the versions that were submitted during the peer review process. They are not the versions of record or the final published versions. They should not be cited or distributed as the published version of this manuscript.

BMJ Open is an open access journal and the full, final, typeset and author-corrected version of record of the manuscript is available on our site with no access controls, subscription charges or pay-per-view fees (http://bmjopen.bmj.com).

If you have any questions on BMJ Open's open peer review process please email info.bmjopen@bmj.com

BMJ Open

ARCHERY: A prospective observational study of artificial intelligence based radiotherapy treatment planning for cervical, head and neck and prostate cancer.

Journal:	*BMJ Open*
Manuscript ID	bmjopen-2023-077253
Article Type:	Protocol
Date Submitted by the Author:	29-Jun-2023

Complete List of Authors:	Aggarwal, Ajay; University College London<br>Court, Laurence Edward; University of Texas, MD Anderson Cancer Center<br>Hoskin, Peter; Mt Vernon Hosp<br>Jacques, Isabella; University College London<br>Kroiss, Mariana; Mt Vernon Hosp<br>Laskar, Sarbani ; Tata Memorial Hospital<br>Lievens, Yolande ; University of Ghent<br>Mallick, Indranil; Tata Memorial Center, Radiation Oncology<br>Abdul Malik, Rozita; University of Malaya<br>Miles, Elizabeth; National Radiotherapy Trials Quality Assurance Group, Mount Vernon Hospital<br>Mohamad, Issa ; King Hussein Cancer Center<br>Murphy, Claire; University College London,<br>Nankivell, Matthew; University College London<br>Parkes, Jeannette; University of Cape Town<br>Parmar, Mahesh; University College London, Institute of Clinical Trials and Methodology - MRC CTU at UCL<br>Roach, Carol; University College London<br>Simonds, Hannah; Stellenbosch University<br>Torode, Julie; King's College London<br>Vanderstraeten, Barbara; University of Ghent<br>Langley, Ruth; University College London Medical School,

Keywords:	RADIOTHERAPY, ONCOLOGY, Adult oncology < ONCOLOGY

SCHOLARONE™
Manuscripts

ARCHERY: A prospective observational study of artificial intelligence based radiotherapy treatment planning for cervical, head and neck and prostate cancer.

Ajay Aggarwal, Laurence Court, Peter Hoskin, Isabella Jacques, Mariana Kroiss, Sarbani Laskar, Yolande Lievens, Indranil Mallick, Rozita Malik, Elizabeth Miles, Issa Mohamed, Claire Murphy, Matthew Nankivell, Jeannette Parkes, Mahesh Parmar, Carol Roach, Hannah Simonds, Julie Torode, Barbara Vanderstraten, Ruth Langley.

For peer review only

ABSTRACT

Introduction

50% of cancer patients require radiotherapy during their disease course, however, only 10-40% of patients in low and middle-income countries (LMICs), have access to it. A shortfall in specialised workforce has been identified as the most significant barrier to expanding radiotherapy capacity. Artificial intelligence (AI) based software has been developed to automate both the delineation of anatomical target structures and the definition of the position, size and shape of the radiation beams. Proposed advantages include improved treatment accuracy, as well as a reduction in the time (from weeks to minutes) and human resources needed to deliver radiotherapy.

Methods

ARCHERY is a non-randomised prospective study to evaluate the quality and economic impact of AI based automated radiotherapy treatment planning for cervical, head and neck, and prostate cancers, which are endemic in LMICs, and for which radiotherapy is the primary curative treatment modality. The sample size of 990 patients (330 for each cancer type) has been calculated based on an estimated 95% treatment plan acceptability rate. Time and cost savings will be analysed as secondary outcome measures using the time-driven activity based costing model. The 48-month study will take place in six public sector cancer hospitals in India (n=2), Jordan (n=1), Malaysia (n=1), and South Africa (n=2) to support implementation of the software in LMICs.

Ethics and Dissemination

The study has received ethical approval from UCL and each of the six study sites. If the study objectives are met, the AI based software will be offered as a not-for-profit web service to public sector state hospitals in LMICs to support expansion of high quality radiotherapy capacity, improving access to and affordability of this key modality of cancer cure and control. A Public and policy engagement plans will involve patients as key partners.

‘Strengths and limitations of this study’

- Study evaluates AI in a broad range of LMICs future roll-out and scale up
- Prospective evaluation of all eligible participants per study site to assess factors influencing selection into study
- Participating centres need Intensity modulated radiotherapy availability with routine use for prostate, cervix and head and neck cancer
- Automated contours and plan will be assessed by double blind peer review according to international quality standards but not directly compared to the manual plan
- Comparison of time savings between automated and manual pathways and the budget impact of integrating AI routinely into the clinical workflow.

INTRODUCTION

By 2030 new cancer cases worldwide are projected to rise to 21.3 million annually, of which approximately 70% will be from low- and middle-income countries (LMICs). Radiotherapy (RT) is a core modality of cancer control and cure for several types of cancer that are common in these settings such as cancers of the cervix, prostate, and head and neck, with 50% of such patients requiring RT during their disease course [1 2]. However, only 10% of patients in low income, and 40% of patients in middle income countries, have access to RT [3]. Limited resources have contributed to long waiting times for treatment resulting in cancer progression, increased morbidity, and inferior survival outcomes, as well as high rates of impoverishing health expenditures [4].

The World Health Organisation (WHO) has set a target, which states that RT is an essential medical device, and should be available to 80% of the world's population by 2025 [5]. It also recognizes that a critical shortfall in the specialised workforce required to deliver RT is the most significant barrier to access. [1] The Global Taskforce on RT for Cancer Control estimated that by 2035, an additional 30,000 radiation oncologists (RO), 22,000 medical physicists (MP) and 80,000 treatment radiographers (RTT) would be required to meet demand.[1]

These workforce shortfalls are coupled with a complex radiotherapy treatment workflow (**Figure 1**), requiring several labour-intensive processes from highly trained people including ROs, MPs, dosimetrists (DOs), and RTTs [6]. The process starts with a dedicated RT computerised tomography (CT) planning scan, followed by the clinician manually segmenting the a. tumour (Gross Tumour Volume (GTV)); b. areas that are at risk of tumour spread (Clinical Target Volumes (CTVs) e.g., regional lymph nodes); organs at risk (OARs) of radiation damage such as the spinal cord, salivary glands, bladder, and rectum. This can take 2-3 hours per patient even in the hands of an experienced practitioner. Next, medical physicists or dosimetrists define the position, size, and shape of the radiation beams to ensure adequate coverage of target structures with radiation at the correct dose which can take up to a further half a day.

For peer review only

Figure 1: Radiotherapy clinical workflow framework for time and motion studies [6]

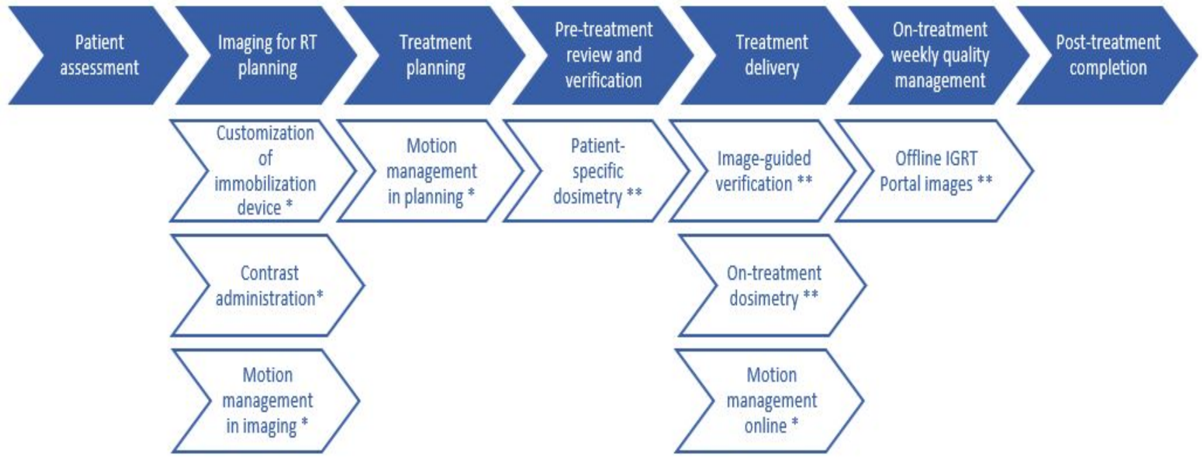

Notes: Full arrows: standard activities. Open arrows: optional activities. *Optional activities defined per tumour site and intent. **optional activities defined based on the technique.

This pre-treatment RT workflow can typically take up to 4 weeks high-income countries and as long as 12 weeks in LMICs due to patient demand and workforce shortages. In addition, errors and delays can accumulate at each stage of RT planning which can negatively impact the quality of treatment. This includes inter-clinician variability in target volume delineation which can have a detrimental impact on patient outcomes [7].

Workforce and skills shortages and lack of financing means countries, have been unable to transition from 2-Dimensional X-ray based planning to CT based methods (e.g. 3D conformal and Intensity Modulated RT (IMRT)), which are associated with better outcomes [8].

The growing demand for RT means that a scalable solution is required to address these challenges. In this regard, a recent WHO report highlighted the potential of digital technologies such as Artificial Intelligence (AI) to contribute to advancing universal health coverage achieving the Sustainable Development Goals such as ensuring equity in access to treatments and their affordability [9].

The Radiotherapy Planning Assistant (RPA) is an AI (Artificial Intelligence) based software that has been developed to automate two key components of the RT planning pathway:

1. Contouring of anatomical areas that are at risk of tumour spread (CTVs) and at risk of radiation damage (OARs).

2. Definition of the position, size, and shape of the radiation beams to the target organs.

The AI-based contouring models that are included in the RPA were developed by the University of Texas MD Anderson Cancer Centre [10 11]. User interfaces and AI-based planning for conformal RT treatments were also developed [12-14], as were in-built quality assurance tools [15 16], and the training and testing of the knowledge-based planning component of the automated planning software which is a function of the Eclipse treatment planning system (Varian Medical Systems). To the best of our knowledge, the RPA is the only application ready for clinical use in head and neck, cervical and prostate cancer which can both contour CTVs and OARs and produce an optimised treatment plan.

The ARCHERY study is a prospective international multi-centre study in India, Jordan, Malaysia, and South Africa to evaluate the quality, time, resource use and cost associated with using the RPA for the treatment of primary cervical, head and neck, and prostate cancers. The first two cancers represent sites which are leading causes of cancer death in LMICs [18 19]. In addition, provision of effective, equitable and affordable RT is vital in order to achieve the WHO's target of eliminating cervical cancer as a public health problem [20]. Prostate cancer represents a very common cancer where RT plays an important curative role [18].

We will assess whether the RPA can automate target volume contouring including OARs and produce RT plans that are able to meet international standards for treatment quality. This would also establish whether AI computer-based software can be routinely integrated into the workflow of diverse high volume LMIC centres which vary in their technical and workforce infrastructure to support the AI integration. The study aims to evaluate the extent to which routine use of the RPA will support better workforce allocation and reduce the costs of radiotherapy treatment delivery. LMIC centres have been chosen in this evaluation as they have the greatest need at present, however the study and its goals are also relevant in the HIC setting and therefore the appraisal of the RPA will use current international quality assurance standards rather than direct comparison of outcomes achieved within individual RT centres [17].

METHODS AND ANALYSIS

PATIENT INCLUSION CRITERIA

1. Patients with:
    a. Histologically confirmed head and neck cancers of the oropharynx, larynx, hypopharynx, and nasopharynx (American Joint Committee on Cancer (AJCC) Stage I-IVB) that have given consent for radical radiotherapy. Induction chemotherapy prior to radiotherapy acceptable.
    OR
    b. Histologically confirmed primary cervical cancer (International Federation of Gynaecology and Obstetrics (FIGO)/AJCC Stage IB-IIIC1) that have

For peer review only

given consent for radical radiotherapy. Induction chemotherapy prior to radiotherapy, or concurrent chemoradiotherapy acceptable.
OR

c. Histologically confirmed primary prostate cancer (AJCC Stage I-IIIC) that have given consent for radical radiotherapy.

2. Provide signed informed consent to participate in the study.
3. Aged ≥ 18years.

PATIENT EXCLUSION CRITERIA

1. Patients requiring radiotherapy after curative surgery or surgery that is intended to remove as much of the tumour as possible.
2. Patients receiving palliative radiotherapy.

Consecutive patients who are eligible will be approached and screening data collected to establish the proportion of eligible patients that consent to participate. Participants are only eligible for the study if they have already provided consent to receive radical radiotherapy and participation in ARCHERY will not change how the patient is treated.

SITE EXCLUSION CRITERIA

1. Centre using exclusively cobalt-based radiotherapy for treatment.
2. Centres not using IMRT or VMAT based radiotherapy planning for head and neck, cervical or prostate cancer.
3. Centres not using standardised international guidelines/protocols for target volume contouring and dosimetric radiation constraints

STUDY CONDUCT

The AI based RT planning tool (the RPA) will be applied to the CT scan acquired as part of a participants planned RT treatment process.

The RPA will be used to automate:

1. CTV and OAR contouring
2. Treatment planning

Randomisation is not required as the initial CT scan of the participant, which is used as the template for contouring and planning, will be used for both the standard and automated approaches. Patients will be treated on the standard plan and will not be affected by the study. Local centres will remain blinded to the results of the automated plans.

For peer review only

CTVs and OAR contouring

Contours will be applied manually to the GTV (for head and neck and cervical cancer) by the treating clinician.

This CT scan with the contoured GTV will be used for the manual and the automated study pathway (Figure 2) to create Plans 1 and 2. The contouring of CTVs and OARs will be performed by the clinician (blue boxes in Figure 2) or the automated by AI using the RPA (orange boxes in Figure 2).

For the prostate cancer pathway there will be no GTV to be outlined but a CTV (prostate gland +/- seminal vesicles) as per current international guidance which will be outlined by the clinician or the RPA.

Figure 2: Study workflow

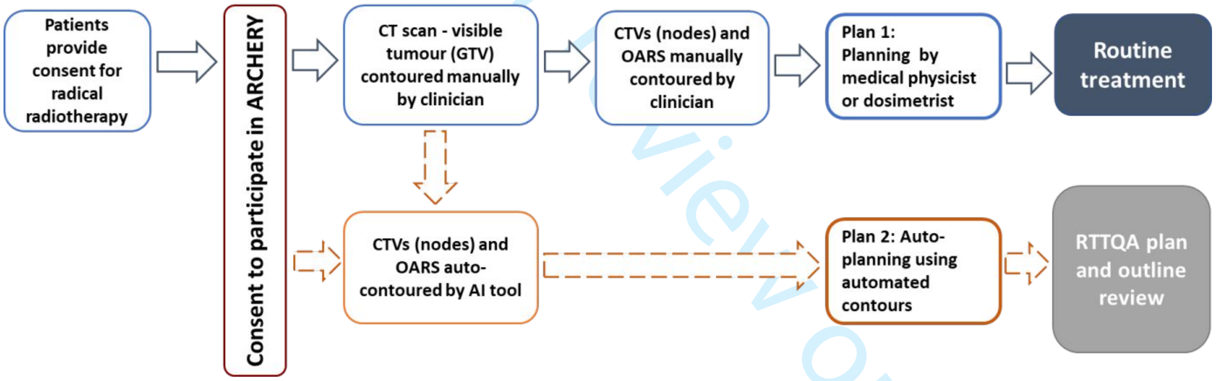

Notes: GTV (Gross tumour volume); CTV (Clinical target volume); OAR (Organs at risk)

Treatment planning

Two plans will be produced for evaluation. Plan 1: Manual contours will undergo planning using standard clinical protocols by a dosimetrist or physicist at the local site, and this will be used to treat the patient (blue box in Figure 2). Plan 2: The automated contours will undergo auto-planning and this will undergo clinical evaluation (orange box in Figure 2).

QUALITY ASSESSMENT PROTOCOL

The final radiotherapy plans will be exported as DICOM® (Digital Imaging and Communication in Medicine) files, to the UK RT Trials Quality Assurance (RTTQA) group who will coordinate the assessment of contouring (CTVs and OARs) and planning (dose distribution) produced by the RPA. An international panel all with greater than 10

For peer review only - http://bmjopen.bmj.com/site/about/guidelines.xhtml

years of experience as a Radiation Oncologist will undertake the evaluation of the treatment contours using a protocol based on international standards for target volume definition and dosimetry.

PROCEDURES FOR ASSESSING EFFICACY

The primary outcome measure is overall treatment plan acceptability, a composite outcome including both the assessment of the delineated CTVs and OARs with reference to internationally accepted contouring guidelines and the adherence to pre-defined dosimetric constraints (Table 1). We will not collect patient outcomes following treatment as important clinical differences in terms of toxicity and tumour control are not expected if these validated criteria are met and patients will not be treated on the automated plans. In addition, variation in the accuracy of patient set up across the centres for treatment may result in important differences in patient outcomes unrelated to the quality of the manual or automated contours [23 24].

The automated plan (Plan 2) will be analysed against the expected plan acceptability rate from the literature which estimates that 95% of RT plans will be acceptable (scores 3 and 4 according to study scoring criteria) for treatment without the requirement for major edits (scores of 1 or 2)[7 25]. Major edits (as defined using the Global Quality Assurance of Radiation Therapy Clinical Trials criteria), are edits that if not undertaken would affect the likelihood of cure, loco-regional control and could result in additional toxicity [26].

The assessment of the contours and dosimetry from the automated plans will be coordinated by the RTTQA group and use international standards and procedures for radiotherapy planning quality assurance which have been agreed by the Global Quality Assurance of Radiation Therapy Clinical Trials Harmonisation Group (GHG) https://rtqaharmonization.org

The RTTQA group will develop procedures and guidelines for OAR and CTV contour assessment in line with international standards for normal tissue and nodal CTVs for cervical, head and neck, and prostate cancer [22 27-31] that will be used by the peer reviewers in their assessments.

A scoring framework developed with reference to GHG protocol variation definitions, will be provided for assessment of contouring with examples given of contours considered clinically not acceptable. The Likert scale below (with potential scope for minor changes) will be used for the grading of contouring:

- Score 4 - ACCEPTABLE – PER PROTOCOL: No edits required. The current contours are clinically acceptable and could be used for treatment without change.

- Score 3 - ACCEPTABLE VARIATION: Minor edits, not expected to have a major clinical impact on treatment outcome. Edits to the current contours should be considered.
- Score 2 - UNACCEPTABLE VARIATION: Major edits, may affect the likelihood of cure, loco-regional control and potentially cause additional toxicity. The current contours are clinically unacceptable.
- Score 1 - UNACCEPTABLE VARIATION WITH TREATMENT MODIFICATION: Major edits, however these variations could not have been reasonably avoided due to clinical necessity as perceived by the treating physician.

Two reviewers will independently review each set of contours. If there is any discordance between the two reviewers i.e., an unacceptable score (1 or 2) by one reviewer and acceptable score (3 or 4) by another, a third reviewer will be consulted, and acceptability will be defined based on a consensus majority rule (2:1).

The dosimetric assessment of the plans will be automated using the auto-extracted Dose Volume Histogram (DVH) constraints. The framework for assessment will be defined for each tumour cohort with reference to GHG criteria [32]. If any of the DVH constraints are not met the plan would be assessed independently by two peer reviewers to evaluate whether there is any mitigation for the dosimetric constraints not being met (e.g., extensive tumour), otherwise the plan will be graded as clinically unacceptable.

Dosimetric constraints will be based on contemporary radiotherapy IMRT RCT protocols for cervical, head and neck, and prostate cancer [28 33 34] but will also require an understanding of differences in protocols used across different regions. The Likert scale below will be used for grading of DVHs:

- Score 4 - ACCEPTABLE – PER PROTOCOL: The radiation therapy treatment was planned according to protocol specifications. *Mandatory and optimal volume planning constraints are met.*
- Score 3 - ACCEPTABLE VARIATION: The radiation therapy treatment was not planned according to all the protocol specifications, but not expected to have a major clinical impact on treatment outcome. *Mandatory volume planning constraints are met but not all the optimal volume planning constraints are achieved.*
- Score 2 - UNACCEPTABLE VARIATION: The radiation therapy treatment was not planned according to protocol specifications and may affect the likelihood of cure, loco-regional control and potentially cause additional toxicity.
- Score 1 - UNACCEPTABLE VARIATION WITH TREATMENT MODIFICATION: The radiation therapy treatment was not planned according to protocol

For peer review only

specifications and may affect the likelihood of cure, loco-regional control and potentially cause additional toxicity. However, these variations could not have been reasonably avoided due to clinical necessity as perceived by the treating physician.

Table 1: Assessment procedures for each treatment plan based on a composite score

Contouring	Dosimetry	Overall Assessment
Score 3 or 4	Score 3 or 4	Acceptable
Score 3 or 4	Score 1 or 2	Unacceptable
Score 1 or 2	Score 3 or 4	Unacceptable
Score 1 or 2	Score 1 or 2	Unacceptable

QUANTIFYING AND MODELLING THE TIME COST SAVINGS FROM USING THE RPA BY USING THE ESTRO APPROVED TIME-DRIVE ACTIVITY-BASED COSTING MODEL

To determine the time savings from the automated approaches compared to the manual approach, time-and-motion studies will be undertaken to populate the ESTRO time-driven activity-based costing model (TD-ABC)[6]. This is considered the most contemporary and robust mechanism for radiation therapy costing and will be used to estimate the cost of delivering RT treatment for each tumour type in each centre, in addition to computing the actual personnel and equipment resource utilisation [6].

The model requires time estimates for all RT processes within the typical RT care pathway including time for contouring anatomy including edits, plan production, peer review, QA checks, plan acceptance and treatment delivery (see Figure 1) [35]. All times will be stratified according to the specialist personnel involved. and undertaken for each of the two study planning pathways outlined in Figure 2. i.e., the 1. standard manual pathway 2. fully automated pathway.

Allied to this will be the collection of detailed information at the centre level of the available resources and their associated costs. This includes:

For peer review only

- Indirect resources: resources (and their costs) that cannot be directly assigned to a single treatment, i.e., personnel, equipment and related infrastructure and maintenance; and overheads.
- Direct material costs related to a particular treatment course (masks, fiducials, contrast material)
- Information on the number of RT courses delivered in the centre (by tumour type, treatment intent, fractionation, complexity)

The outputs from the workflow timings study and data collection on the resources available and courses delivered at each individual centre will be key inputs into the model to estimate differences in costs and resource needs for cervical cancer, head and neck cancer and prostate cancer treatment courses using the two approaches (fully automated versus standard).

Using this information, we will be able to assess the impact on resource needs and associated costs (in particular for specialist personnel e.g. ROs from scaling up automation to other cancer types, and undertake cost-minimisation and budget impact analyses [36]. The costing and cost-saving calculations will be performed at the level of each country and centre , The budget impact analysis will inform the affordability of the intervention to the health system, in terms of the feasibility of expanding the number of patients that can be treated with RT[37].

SAMPLE SIZE

The sample size for each tumour population has been calculated based on an overall 95% plan acceptability rate, with a confidence interval which is no wider than +/- 5% in absolute terms around this figure This rate is considered necessary to support implementation of the software as the clinical community would be unlikely to accept a lower plan acceptability rate of, for example 80%.

To achieve at least 90% power that the lower confidence limit for the acceptability plan will be greater than 90%, assuming alpha=0.05 (or one-sided 0.025) and a binomial distribution, the required sample size for is approximately 330 for each tumour population [38].

STATISTICAL ANALYSIS PLAN

We will fit a logistic regression model to the binary outcome of plan acceptability adjusted by centre with cluster robust standard errors. We calculate the overall marginal proportion estimate balanced for centre, to calculate the robust confidence interval that maintains the family-wise error rate (FWER) at 0.05. An additional analysis will investigate the inter-rater agreement for the plan. For each cancer this will be assessed by calculating the kappa statistic between the first 2 expert raters assessing plans, so excluding the conditional 3rd rating.

For peer review only - http://bmjopen.bmj.com/site/about/guidelines.xhtml

COMPLETION OF PARTICIPANT FOLLOW UP

Participants have no further follow up after their radiotherapy CT planning scan. Clinical management is as per the centre's standard of care.

PATIENT AND PUBLIC INVOLVEMENT

Patient partners present at the workshops have directly informed the research question, and cancer types chosen. The need for inclusion of AI and this study across LMIC partners has been defined through three international global RT workshops between 2017 and 2019, attended by the Chief Investigator (AA) at CERN (The European Organization for Nuclear Research), and in the UK and Botswana. As well as patient partners, attendees included clinical, academic, and policy stakeholders from LMICs, including Ghana, Botswana, South Africa, Nigeria, Zambia, and Jordan

With respect to study design, patient partners have highlighted that patient follow up in LMICs can be challenging, with very low rates of follow up following treatment. This has therefore informed the primary outcome measure of "radiotherapy plan acceptability" as a measure of treatment quality without the necessity for ongoing follow up. Patients will be treated using the current standard of care to avoid any delays, with the appraisal of automated plans occurring offline but using the RT CT planning scan that is acquired as part of the standard treatment process

Patients will be involved in the dissemination strategy. The patient-involvement coordinator (JT), will work with community engagement and involvement partners in each of the participating countries to develop a programme for dissemination of results.

Ethics and dissemination

The protocol has received ethical approval from UCL Ethics and each of the six recruiting sites in India, Jordan, Malaysia and South Africa. International sites will comply with the principles of GCP as laid down by the ICH topic E6 (R2) and other applicable national regulations

Although there will be no clinical impact of the intervention or risk to the patient as they will continue to be treated according to the standard treatment pathway and not receive any additional imaging intervention or treatment, protections have been put in place for the use of pseudonymised CT scans and treatment planning details stored as DICOM® files. Patients will be enrolled with written informed consent.

DISSEMINATION

If the study objectives are met, the AI based software will be offered as a not-for-profit web service to public sector state hospitals in LMICs. We anticipate this will support

For peer review only - http://bmjopen.bmj.com/site/about/guidelines.xhtml

rapid adoption into daily practice and facilitate expansion of high-quality radiotherapy in these countries. This in turn will improve access, and affordability of this critical treatment option and therefore also improve survival and the quality of life of patients with cancer globally.

REFERENCES

1. Atun R, Jaffray DA, Barton MB, et al. Expanding global access to radiotherapy. *The Lancet Oncology* 2015;16(10):1153-86. doi: 10.1016/S1470-2045(15)00222-3
2. Delaney GP, Barton MB. Evidence-based estimates of the demand for radiotherapy. *Clin Oncol (R Coll Radiol)* 2015;27(2):70-6. doi: 10.1016/j.clon.2014.10.005 [published Online First: 2014/12/03]
3. Zubizarreta EH, Fidarova E, Healy B, et al. Need for radiotherapy in low and middle income countries - the silent crisis continues. *Clin Oncol (R Coll Radiol)* 2015;27(2):107-14. doi: 10.1016/j.clon.2014.10.006 [published Online First: 2014/12/03]
4. Anakwenze CP, Ntekim A, Trock B, et al. Barriers to radiotherapy access at the University College Hospital in Ibadan, Nigeria. *Clin Transl Radiat Oncol* 2017;5:1-5. doi: 10.1016/j.ctro.2017.05.003 [published Online First: 2018/03/30]
5. Berumen AV. Access to medical devices for Universal Health Coverage and achieve of SDGs: World Health Organisation, 2019.
6. Defourny N, Perrier L, Borras JM, et al. National costs and resource requirements of external beam radiotherapy: A time-driven activity-based costing model from the ESTRO-HERO project. *Radiotherapy and oncology : journal of the European Society for Therapeutic Radiology and Oncology* 2019;138:187-94. doi: 10.1016/j.radonc.2019.06.015 [published Online First: 2019/07/19]
7. Cox S, Cleves A, Clementel E, et al. Impact of deviations in target volume delineation - Time for a new RTQA approach? *Radiotherapy and oncology : journal of the European Society for Therapeutic Radiology and Oncology* 2019;137:1-8. doi: 10.1016/j.radonc.2019.04.012 [published Online First: 2019/05/01]
8. Rodin D, Tawk B, Mohamad O, et al. Hypofractionated radiotherapy in the real-world setting: An international ESTRO-GIRO survey. *Radiotherapy and oncology : journal of the European Society for Therapeutic Radiology and Oncology* 2021;157:32-39. doi: 10.1016/j.radonc.2021.01.003 [published Online First: 2021/01/17]
9. WHO. WHO guideline: recommendations on digital interventions for health system strengthening, 2019.
10. Rhee DJ, Cardenas CE, Elhalawani H, et al. Automatic detection of contouring errors using convolutional neural networks. *Medical Physics* 2019;46(11):5086-97. doi: 10.1002/mp.13814
11. Cardenas CE, Anderson BM, Aristophanous M, et al. Auto-delineation of oropharyngeal clinical target volumes using 3D convolutional neural networks. *Phys Med Biol* 2018;63(21):215026. doi: 10.1088/1361-6560/aae8a9 [published Online First: 2018/11/08]

For peer review only

12. Kisling K, Zhang L, Simonds H, et al. Fully Automatic Treatment Planning for External-Beam Radiation Therapy of Locally Advanced Cervical Cancer: A Tool for Low-Resource Clinics. *J Glob Oncol* 2019(5):1-9. doi: 10.1200/jgo.18.00107
13. McCarroll RE, Beadle BM, Balter PA, et al. Retrospective Validation and Clinical Implementation of Automated Contouring of Organs at Risk in the Head and Neck: A Step Toward Automated Radiation Treatment Planning for Low- and Middle-Income Countries. *J Glob Oncol* 2018(4):1-11. doi: 10.1200/jgo.18.00055
14. Court LE, Kisling K, McCarroll R, et al. Radiation Planning Assistant - A Streamlined, Fully Automated Radiotherapy Treatment Planning System. *J Vis Exp* 2018(134) doi: 10.3791/57411 [published Online First: 2018/05/01]
15. Kisling K, Cardenas C, Anderson BM, et al. Automatic Verification of Beam Apertures for Cervical Cancer Radiation Therapy. *Practical radiation oncology* 2020;10(5):e415-e24. doi: 10.1016/j.prro.2020.05.001 [published Online First: 2020/05/26]
16. Rhee DJ, Cardenas CE, Elhalawani H, et al. Automatic detection of contouring errors using convolutional neural networks. *Med Phys* 2019;46(11):5086-97. doi: 10.1002/mp.13814 [published Online First: 2019/09/11]
17. Lievens Y, Defourny N, Coffey M, et al. Radiotherapy staffing in the European countries: final results from the ESTRO-HERO survey. *Radiotherapy and oncology : journal of the European Society for Therapeutic Radiology and Oncology* 2014;112(2):178-86. doi: 10.1016/j.radonc.2014.08.034 [published Online First: 2014/10/11]
18. Sung H, Ferlay J, Siegel RL, et al. Global Cancer Statistics 2020: GLOBOCAN Estimates of Incidence and Mortality Worldwide for 36 Cancers in 185 Countries. *CA Cancer J Clin* 2021;71(3):209-49. doi: 10.3322/caac.21660 [published Online First: 2021/02/05]
19. Arbyn M, Weiderpass E, Bruni L, et al. Estimates of incidence and mortality of cervical cancer in 2018: a worldwide analysis. *The Lancet Global Health* 2020;8(2):e191-e203. doi: 10.1016/S2214-109X(19)30482-6
20. Organization WH. Global strategy to accelerate the eliminaton of cervical cancer as a public health problem 2020.
21. Chow LQM. Head and Neck Cancer. *The New England journal of medicine* 2020;382(1):60-72. doi: 10.1056/NEJMra1715715 [published Online First: 2020/01/02]
22. Brouwer CL, Steenbakkers RJ, Bourhis J, et al. CT-based delineation of organs at risk in the head and neck region: DAHANCA, EORTC, GORTEC, HKNPCSG, NCIC CTG, NCRI, NRG Oncology and TROG consensus guidelines. *Radiotherapy and oncology : journal of the European Society for Therapeutic Radiology and Oncology* 2015;117(1):83-90. doi: 10.1016/j.radonc.2015.07.041 [published Online First: 2015/08/19]
23. Yeung TK, Bortolotto K, Cosby S, et al. Quality assurance in radiotherapy: evaluation of errors and incidents recorded over a 10 year period. *Radiotherapy and oncology : journal of the European Society for Therapeutic Radiology and Oncology* 2005;74(3):283-91. doi: 10.1016/j.radonc.2004.12.003 [published Online First: 2005/03/15]

For peer review only

24. Shafiq J, Barton M, Noble D, et al. An international review of patient safety measures in radiotherapy practice. *Radiotherapy and Oncology* 2009;92(1):15-21. doi: https://doi.org/10.1016/j.radonc.2009.03.007
25. Brunskill K, Nguyen TK, Boldt RG, et al. Does Peer Review of Radiation Plans Affect Clinical Care? A Systematic Review of the Literature. *Int J Radiat Oncol Biol Phys* 2017;97(1):27-34. doi: 10.1016/j.ijrobp.2016.09.015 [published Online First: 2016/11/07]
26. Lewis PJ, Court LE, Lievens Y, et al. Structure and Processes of Existing Practice in Radiotherapy Peer Review: A Systematic Review of the Literature. *Clin Oncol (R Coll Radiol)* 2021;33(4):248-60. doi: 10.1016/j.clon.2020.10.017 [published Online First: 2020/11/09]
27. Grégoire V, Ang K, Budach W, et al. Delineation of the neck node levels for head and neck tumors: a 2013 update. DAHANCA, EORTC, HKNPCSG, NCIC CTG, NCRI, RTOG, TROG consensus guidelines. *Radiotherapy and oncology : journal of the European Society for Therapeutic Radiology and Oncology* 2014;110(1):172-81. doi: 10.1016/j.radonc.2013.10.010 [published Online First: 2013/11/05]
28. Tanderup K. PR, Lindegaard J., Kiristis C, Juergenliemk-Schulz I., Leeuw A., Liederer T., Tan L. Image guided intensity modulated External beam radiochemotherapy and MRI based Brachytherapy in locally advanced Cervical Cancer. EMBRACE-II 2015 [Available from: https://www.embracestudy.dk/UserUpload/PublicDocuments/EMBRACE%20II%20Protocol.pdf.
29. Salembier C, Villeirs G, De Bari B, et al. ESTRO ACROP consensus guideline on CT- and MRI-based target volume delineation for primary radiation therapy of localized prostate cancer. *Radiotherapy and oncology : journal of the European Society for Therapeutic Radiology and Oncology* 2018;127(1):49-61. doi: 10.1016/j.radonc.2018.01.014 [published Online First: 2018/03/03]
30. Mir R, Kelly SM, Xiao Y, et al. Organ at risk delineation for radiation therapy clinical trials: Global Harmonization Group consensus guidelines. *Radiotherapy and oncology : journal of the European Society for Therapeutic Radiology and Oncology* 2020;150:30-39. doi: 10.1016/j.radonc.2020.05.038 [published Online First: 2020/06/07]
31. NRG Oncology. Gynecologic Cancer Atlases, Templates, & Tools 2022 [Available from: https://www.nrgoncology.org/ciro-gynecologic.
32. Hansen CR, Crijns W, Hussein M, et al. Radiotherapy Treatment plannINg study Guidelines (RATING): A framework for setting up and reporting on scientific treatment planning studies. *Radiotherapy and oncology : journal of the European Society for Therapeutic Radiology and Oncology* 2020;153:67-78. doi: 10.1016/j.radonc.2020.09.033 [published Online First: 2020/09/26]
33. Dearnaley D, Syndikus I, Mossop H, et al. Conventional versus hypofractionated high-dose intensity-modulated radiotherapy for prostate cancer: 5-year outcomes of the randomised, non-inferiority, phase 3 CHHiP trial. *The Lancet Oncology* 2016;17(8):1047-60. doi: 10.1016/S1470-2045(16)30102-4
34. Stelmes JJ, Vu E, Grégoire V, et al. Quality assurance of radiotherapy in the ongoing EORTC 1420 "Best of" trial for early stage oropharyngeal, supraglottic

For peer review only

and hypopharyngeal carcinoma: results of the benchmark case procedure. *Radiat Oncol* 2021;16(1):81. doi: 10.1186/s13014-021-01809-2 [published Online First: 2021/05/03]
35. Lievens Y, Obyn C, Mertens AS, et al. Stereotactic body radiotherapy for lung cancer: how much does it really cost? *Journal of thoracic oncology : official publication of the International Association for the Study of Lung Cancer* 2015;10(3):454-61. doi: 10.1097/jto.0000000000000421 [published Online First: 2014/11/08]
36. Lievens YRD, Aggarwal A. Financial and Economic Considerations in Radiation Oncology. In: Dyk JV, ed. The Modern Technology of Radiation Oncology. United States of America: Medical Physics Publishing 2020.
37. Van Dyk J, Zubizarreta E, Lievens Y. Cost evaluation to optimise radiation therapy implementation in different income settings: A time-driven activity-based analysis. *Radiotherapy and oncology : journal of the European Society for Therapeutic Radiology and Oncology* 2017;125(2):178-85. doi: 10.1016/j.radonc.2017.08.021 [published Online First: 2017/09/28]
38. Ruvuna F. Unequal Center Sizes, Sample Size, and Power in Multicenter Clinical Trials. *Drug Information Journal* 2004;38(4):387-94. doi: 10.1177/009286150403800409

Authors' contributions

AA and LC conceived the original study idea. The research design was developed in conjunction with methodological experts at UCL – MP and RL; Ghent University – YL and BV and the Radiotherapy trials quality assurance group – PH, MK, EM and the study site clinical lead investigators (SL, IM, RM, JP, HS, IM). The protocol was then further developed including study conduct, data management statistical evaluation with additional contributions from UCL – CM, MN, CR, IJ.

Funding statement: 'This work was supported by National Institutes of Health (NIH) National Cancer Institute (NCI) (Award No. 1U01CA269143-01) and the Rising Tide Foundation (Award No. CCR-21-300) will be providing funding for the ARCHERY study. Additional support will be provided by Medical Research Council, via UK Research and Innovation (UKRI) (core funding - MC_UU_00004/01 - Programme 1)

For peer review only

SPIRIT
STANDARD PROTOCOL ITEMS: RECOMMENDATIONS FOR INTERVENTIONAL TRIALS

SPIRIT 2013 Checklist: Recommended items to address in a clinical trial protocol and related documents*

Section/item	Item No	Description	Page Number
**Administrative information**			
Title	1	Descriptive title identifying the study design, population, interventions, and, if applicable, trial acronym	Page IV, summary of the study
Trial registration	2a	Trial identifier and registry name. If not yet registered, name of intended registry	Page IV – ISRCTN and Clinicaltrials.gov
	2b	All items from the World Health Organization Trial Registration Data Set	Pages II - IV
Protocol version	3	Date and version identifier	Page IV, summary of study
Funding	4	Sources and types of financial, material, and other support	Page IV, summary of study
Roles and responsibilities	5a	Names, affiliations, and roles of protocol contributors	Page III, trial administration
	5b	Name and contact information for the trial sponsor	Page II under “Sponsor”
	5c	Role of study sponsor and funders, if any, in study design; collection, management, analysis, and interpretation of data; writing of the report; and the decision to submit the report for publication, including whether they will have ultimate authority over any of these activities	Page 30 – section 13.4
	5d	Composition, roles, and responsibilities of the coordinating centre, steering committee, endpoint adjudication committee, data management team, and other individuals or groups overseeing the trial, if applicable (see Item 21a for data monitoring committee)	Page 30, oversight and trial committees.
**Introduction**			

For peer review only

Background and rationale	6a	Description of research question and justification for undertaking the trial, including summary of relevant studies (published and unpublished) examining benefits and harms for each intervention	Please see Pages 1 to 5
	6b	Explanation for choice of comparators	
Objectives	7	Specific objectives or hypotheses	Page 7.
Trial design	8	Description of trial design including type of trial (eg, parallel group, crossover, factorial, single group), allocation ratio, and framework (eg, superiority, equivalence, noninferiority, exploratory)	Page IV and Pages 14 and 15

Methods: Participants, interventions, and outcomes

Study setting	9	Description of study settings (eg, community clinic, academic hospital) and list of countries where data will be collected. Reference to where list of study sites can be obtained	Please see Page 8
Eligibility criteria	10	Inclusion and exclusion criteria for participants. If applicable, eligibility criteria for study centres and individuals who will perform the interventions (eg, surgeons, psychotherapists)	Please see Page 8 and 9 for study site inclusion/exclusion criteria. Please see Page 11 regarding selection of participants.
Interventions	11a	Interventions for each group with sufficient detail to allow replication, including how and when they will be administered	Please see Page 14 and 15
	11b	Criteria for discontinuing or modifying allocated interventions for a given trial participant (eg, drug dose change in response to harms, participant request, or improving/worsening disease)	Please see Page 15
	11c	Strategies to improve adherence to intervention protocols, and any procedures for monitoring adherence (eg, drug tablet return, laboratory tests)	N/A
	11d	Relevant concomitant care and interventions that are permitted or prohibited during the trial	N/A

For peer review only - http://bmjopen.bmj.com/site/about/guidelines.xhtml

Outcomes	12	Primary, secondary, and other outcomes, including the specific measurement variable (eg, systolic blood pressure), analysis metric (eg, change from baseline, final value, time to event), method of aggregation (eg, median, proportion), and time point for each outcome. Explanation of the clinical relevance of chosen efficacy and harm outcomes is strongly recommended	Please see page 16 and 17. Please also see Page 20 and 21.
Participant timeline	13	Time schedule of enrolment, interventions (including any run-ins and washouts), assessments, and visits for participants. A schematic diagram is highly recommended (see Figure)	Please see Page 14
Sample size	14	Estimated number of participants needed to achieve study objectives and how it was determined, including clinical and statistical assumptions supporting any sample size calculations	Please see Page 21
Recruitment	15	Strategies for achieving adequate participant enrolment to reach target sample size	Recruitment not expected to be a challenge given numbers of cases treated per year in each centre and that the study does not result in any deviation from the standard treatment plan of the patient
**Methods: Assignment of interventions (for controlled trials)**			**Not Applicable**
Allocation:			
Sequence generation	16a	Method of generating the allocation sequence (eg, computer-generated random numbers), and list of any factors for stratification. To reduce predictability of a random sequence, details of any planned restriction (eg, blocking) should be provided in a separate document that is unavailable to those who enrol participants or assign interventions	N/A

Allocation concealment mechanism	16b	Mechanism of implementing the allocation sequence (eg, central telephone; sequentially numbered, opaque, sealed envelopes), describing any steps to conceal the sequence until interventions are assigned	N/A
Implementation	16c	Who will generate the allocation sequence, who will enrol participants, and who will assign participants to interventions	N/A
Blinding (masking)	17a	Who will be blinded after assignment to interventions (eg, trial participants, care providers, outcome assessors, data analysts), and how	N/A
	17b	If blinded, circumstances under which unblinding is permissible, and procedure for revealing a participant's allocated intervention during the trial	N/A
**Methods: Data collection, management, and analysis**			
Data collection methods	18a	Plans for assessment and collection of outcome, baseline, and other trial data, including any related processes to promote data quality (eg, duplicate measurements, training of assessors) and a description of study instruments (eg, questionnaires, laboratory tests) along with their reliability and validity, if known. Reference to where data collection forms can be found, if not in the protocol	Please see Page 19.
	18b	Plans to promote participant retention and complete follow-up, including list of any outcome data to be collected for participants who discontinue or deviate from intervention protocols	Please see Page 15. No concerns re: retention as no follow up required
Data management	19	Plans for data entry, coding, security, and storage, including any related processes to promote data quality (eg, double data entry; range checks for data values). Reference to where details of data management procedures can be found, if not in the protocol	Please see Pages 24-27 which focuses on data collection and storage of the different data types.

Statistical methods	20a	Statistical methods for analysing primary and secondary outcomes. Reference to where other details of the statistical analysis plan can be found, if not in the protocol	Please see Page 22 for statistical methods for analysing our primary outcome measure (plan acceptability). Our secondary outcome measure – cost savings will be done using the Time driven costing model. Please see Page 20 and 21.
	20b	Methods for any additional analyses (eg, subgroup and adjusted analyses)	N/A
	20c	Definition of analysis population relating to protocol non-adherence (eg, as randomised analysis), and any statistical methods to handle missing data (eg, multiple imputation)	N/A
**Methods: Monitoring**			
Data monitoring	21a	Composition of data monitoring committee (DMC); summary of its role and reporting structure; statement of whether it is independent from the sponsor and competing interests; and reference to where further details about its charter can be found, if not in the protocol. Alternatively, an explanation of why a DMC is not needed	Due to the nature of this particular study we will not have a DMC but a study steering committee.
	21b	Description of any interim analyses and stopping guidelines, including who will have access to these interim results and make the final decision to terminate the trial	No interim analyses planned. We will assure the quality of the data coming in.
Harms	22	Plans for collecting, assessing, reporting, and managing solicited and spontaneously reported adverse events and other unintended effects of trial interventions or trial conduct	Not applicable as there is no intervention received by the patient as part of the study
Auditing	23	Frequency and procedures for auditing trial conduct, if any, and whether the process will be independent from investigators and the sponsor	Section 7
**Ethics and dissemination**			

Research ethics approval	24	Plans for seeking research ethics committee/institutional review board (REC/IRB) approval	REC/IRB approvals are required for each site. Please see Page 26
Protocol amendments	25	Plans for communicating important protocol modifications (eg, changes to eligibility criteria, outcomes, analyses) to relevant parties (eg, investigators, REC/IRBs, trial participants, trial registries, journals, regulators)	Please see Page 36
Consent or assent	26a	Who will obtain informed consent or assent from potential trial participants or authorised surrogates, and how (see Item 32)	Please see Page 24 under ethical conduct. Page 8 highlights the investigators ability to create a delegate log so that appropriately qualified persons can take consent.
	26b	Additional consent provisions for collection and use of participant data and biological specimens in ancillary studies, if applicable	N/A
Confidentiality	27	How personal information about potential and enrolled participants will be collected, shared, and maintained in order to protect confidentiality before, during, and after the trial	Please see Page 25 and 26.
Declaration of interests	28	Financial and other competing interests for principal investigators for the overall trial and each study site	Please see PI qualifications and agreements P8
Access to data	29	Statement of who will have access to the final trial dataset, and disclosure of contractual agreements that limit such access for investigators	Please see Page 35.
Ancillary and post-trial care	30	Provisions, if any, for ancillary and post-trial care, and for compensation to those who suffer harm from trial participation	See section on indemnity Page 28.

For peer review only

Dissemination policy	31a	Plans for investigators and sponsor to communicate trial results to participants, healthcare professionals, the public, and other relevant groups (eg, via publication, reporting in results databases, or other data sharing arrangements), including any publication restrictions	Please see Pages 33 and 34
	31b	Authorship eligibility guidelines and any intended use of professional writers	
	31c	Plans, if any, for granting public access to the full protocol, participant-level dataset, and statistical code	
**Appendices**			
Informed consent materials	32	Model consent form and other related documentation given to participants and authorised surrogates	Please PIS included with submission.
Biological specimens	33	Plans for collection, laboratory evaluation, and storage of biological specimens for genetic or molecular analysis in the current trial and for future use in ancillary studies, if applicable	No ancillary studies planned.

*It is strongly recommended that this checklist be read in conjunction with the SPIRIT 2013 Explanation & Elaboration for important clarification on the items. Amendments to the protocol should be tracked and dated. The SPIRIT checklist is copyrighted by the SPIRIT Group under the Creative Commons "Attribution-NonCommercial-NoDerivs 3.0 Unported" license.

For peer review only

BMJ Open

ARCHERY: A prospective observational study of artificial intelligence based radiotherapy treatment planning for cervical, head and neck and prostate cancer - study protocol

Journal:	*BMJ Open*
Manuscript ID	bmjopen-2023-077253.R1
Article Type:	Protocol
Date Submitted by the Author:	18-Sep-2023

Complete List of Authors:	Aggarwal, Ajay; University College London<br>Court, Laurence Edward; University of Texas, MD Anderson Cancer Center<br>Hoskin, Peter; Mt Vernon Hosp<br>Jacques, Isabella; University College London<br>Kroiss, Mariana; Mt Vernon Hosp<br>Laskar, Sarbani ; Tata Memorial Hospital<br>Lievens, Yolande ; University of Ghent<br>Mallick, Indranil; Tata Memorial Center, Radiation Oncology<br>Abdul Malik, Rozita; University of Malaya<br>Miles, Elizabeth; National Radiotherapy Trials Quality Assurance Group, Mount Vernon Hospital<br>Mohamad, Issa ; King Hussein Cancer Center<br>Murphy, Claire; University College London,<br>Nankivell, Matthew; University College London<br>Parkes, Jeannette; University of Cape Town<br>Parmar, Mahesh; University College London, Institute of Clinical Trials and Methodology - MRC CTU at UCL<br>Roach, Carol; University College London<br>Simonds, Hannah; Stellenbosch University<br>Torode, Julie; King's College London<br>Vanderstraeten, Barbara; University of Ghent<br>Langley, Ruth; University College London Medical School,

**Primary Subject Heading**:	Oncology
Secondary Subject Heading:	Radiology and imaging
Keywords:	RADIOTHERAPY, ONCOLOGY, Adult oncology < ONCOLOGY

SCHOLARONE™ Manuscripts

ARCHERY: A prospective observational study of artificial intelligence based radiotherapy treatment planning for cervical, head and neck and prostate cancer - study protocol

Ajay Aggarwal[1,2*], Laurence Court[3], Peter Hoskin[4], Isabella Jacques[1], Mariana Kroiss[4], Sarbani Laskar[5], Yolande Lievens[6], Indranil Mallick[7], Rozita Malik[8], Elizabeth Miles[4], Issa Mohamad[9], Claire Murphy[1], Matthew Nankivell[1], Jeannette Parkes[10], Mahesh Parmar[1], Carol Roach[1], Hannah Simonds[11], Julie Torode[12], Barbara Vanderstraeten[6], Ruth Langley[1].

1 MRC Clinical Trials Unit at UCL, Institute of Clinical Trials & Methodology, London, UK

2 London School of Hygiene and Tropical Medicine, UK

3 MD Anderson Cancer Center, University of Texas, USA

4 Mount Vernon Cancer Centre, Middlesex, UK

5 Tata Memorial Hospital, Mumbai, India

6 Ghent University, Belgium

7 Tata Medical Center, Kolkata, India

8 University Malaya Medical Centre, Malaysia

9 King Hussein Cancer Centre, Jordan

10 University of Cape Town, South Africa

11 Stellenbosch University, South Africa

12 Institute of Cancer Policy, King's College London, UK

* Corresponding author
a.aggarwal@ucl.ac.uk

For peer review only

ABSTRACT

Introduction

50% of cancer patients require radiotherapy during their disease course, however, only 10-40% of patients in low and middle-income countries (LMICs), have access to it. A shortfall in specialised workforce has been identified as the most significant barrier to expanding radiotherapy capacity. Artificial intelligence (AI) based software has been developed to automate both the delineation of anatomical target structures and the definition of the position, size and shape of the radiation beams. Proposed advantages include improved treatment accuracy, as well as a reduction in the time (from weeks to minutes) and human resources needed to deliver radiotherapy.

Methods

ARCHERY is a non-randomised prospective study to evaluate the quality and economic impact of AI based automated radiotherapy treatment planning for cervical, head and neck, and prostate cancers, which are endemic in LMICs, and for which radiotherapy is the primary curative treatment modality. The sample size of 990 patients (330 for each cancer type) has been calculated based on an estimated 95% treatment plan acceptability rate. Time and cost savings will be analysed as secondary outcome measures using the time-driven activity based costing model. The 48-month study will take place in six public sector cancer hospitals in India (n=2), Jordan (n=1), Malaysia (n=1), and South Africa (n=2) to support implementation of the software in LMICs.

Ethics and Dissemination

The study has received ethical approval from UCL and each of the six study sites. If the study objectives are met, the AI based software will be offered as a not-for-profit web service to public sector state hospitals in LMICs to support expansion of high quality radiotherapy capacity, improving access to and affordability of this key modality of cancer cure and control. A Public and policy engagement plans will involve patients as key partners.

'Strengths and limitations of this study'

- Study evaluates AI in a broad range of LMICs future roll-out and scale up
- Prospective evaluation of all eligible participants per study site to assess factors influencing selection into study
- Participating centres need Intensity modulated radiotherapy availability with routine use for prostate, cervix and head and neck cancer
- Automated contours and plan will be assessed by double blind peer review according to international quality standards but not directly compared to the manual plan
- Comparison of time savings between automated and manual pathways and the budget impact of integrating AI routinely into the clinical workflow.

INTRODUCTION

By 2030 new cancer cases worldwide are projected to rise to 21.3 million annually, of which approximately 70% will be from low- and middle-income countries (LMICs). Radiotherapy (RT) is a core modality of cancer control and cure for several types of cancer that are common in these settings such as cancers of the cervix, prostate, and head and neck, with 50% of such patients requiring RT during their disease course (1, 2). However, only 10% of patients in low income, and 40% of patients in middle income countries, have access to RT (3). Limited resources have contributed to long waiting times for treatment resulting in cancer progression, increased morbidity, and inferior survival outcomes, as well as high rates of impoverishing health expenditures (4).

The World Health Organisation (WHO) has set a target, which states that RT is an essential medical device, and should be available to 80% of the world's population by 2025 (5). It also recognizes that a critical shortfall in the specialised workforce required to deliver RT is the most significant barrier to access. (1) The Global Taskforce on RT for Cancer Control estimated that by 2035, an additional 30,000 radiation oncologists (RO), 22,000 medical physicists (MP) and 80,000 treatment radiographers (RTT) would be required to meet demand.(1)

These workforce shortfalls are coupled with a complex radiotherapy treatment workflow, requiring several labour-intensive processes from highly trained people including ROs, MPs, dosimetrists (DOs), and RTTs (6). The process starts with a dedicated RT computerised tomography (CT) planning scan, followed by the clinician manually segmenting the a. tumour (Gross Tumour Volume (GTV)); b. areas that are at risk of tumour spread (Clinical Target Volumes (CTVs) e.g., regional lymph nodes); organs at risk (OARs) of radiation damage such as the spinal cord, salivary glands, bladder, and rectum. This can take 2-3 hours per patient even in the hands of an experienced practitioner. Next, medical physicists or dosimetrists define the position, size, and shape of the radiation beams to ensure adequate coverage of target structures with radiation at the correct dose which can take up to a further half a day.

This pre-treatment RT workflow can typically take up to 4 weeks high-income countries and as long as 12 weeks in LMICs due to patient demand and workforce shortages. In addition, errors and delays can accumulate at each stage of RT planning which can negatively impact the quality of treatment. This includes inter-clinician variability in target volume delineation which can have a detrimental impact on patient outcomes (7).

Workforce and skills shortages and lack of financing means countries, have been unable to transition from 2-Dimensional X-ray based planning to CT based methods (e.g. 3D conformal and Intensity Modulated RT (IMRT)), which are associated with better outcomes (8).

The growing demand for RT means that a scalable solution is required to address these challenges. In this regard, a recent WHO report highlighted the potential of digital technologies such as Artificial Intelligence (AI) to contribute to advancing universal health coverage achieving the Sustainable Development Goals such as ensuring equity in access to treatments and their affordability (9).

The Radiotherapy Planning Assistant (RPA) is an AI (Artificial Intelligence) based software that has been developed to automate two key components of the RT planning pathway:

1. Contouring of anatomical areas that are at risk of tumour spread (CTVs) and at risk of radiation damage (OARs).

2. Definition of the position, size, and shape of the radiation beams to the target organs.

The AI-based contouring models that are included in the RPA were developed by the University of Texas MD Anderson Cancer Centre (10, 11). User interfaces and AI-based planning for conformal RT treatments were also developed (12-14), as were in-built quality assurance tools (15, 16), and the training and testing of the knowledge-based planning component of the automated planning software which is a function of the Eclipse treatment planning system (Varian Medical Systems). To the best of our knowledge, the RPA is the only application ready for clinical use in head and neck, cervical and prostate cancer which can both contour CTVs and OARs and produce an optimised treatment plan.

The ARCHERY study is a prospective international multi-centre study in India, Jordan, Malaysia, and South Africa to evaluate the quality, time, resource use and cost associated with using the RPA for the treatment of primary cervical, head and neck, and prostate cancers. The first two cancers represent sites which are leading causes of cancer death in LMICs (17, 18). In addition, provision of effective, equitable and affordable RT is vital in order to achieve the WHO's target of eliminating cervical cancer as a public health problem (19). Prostate cancer represents a very common cancer where RT plays an important curative role (17).

For peer review only

We will assess whether the RPA can automate target volume contouring including OARs and produce RT plans that are able to meet international standards for treatment quality. This would also establish whether AI computer-based software can be routinely integrated into the workflow of diverse high volume LMIC centres which vary in their technical and workforce infrastructure to support the AI integration. The study aims to evaluate the extent to which routine use of the RPA will support better workforce allocation and reduce the costs of radiotherapy treatment delivery. LMIC centres have been chosen in this evaluation as they have the greatest need at present, however the study and its goals are also relevant in the HIC setting and therefore the appraisal of the RPA will use current international quality assurance standards rather than direct comparison of outcomes achieved within individual RT centres (20).

METHODS AND ANALYSIS

PATIENT INCLUSION CRITERIA

1. Patients with:
   a. Histologically confirmed head and neck cancers of the oropharynx, larynx, hypopharynx, and nasopharynx (American Joint Committee on Cancer (AJCC) Stage I-IVB) that have given consent for radical radiotherapy. Induction chemotherapy prior to radiotherapy acceptable.
   OR
   b. Histologically confirmed primary cervical cancer (International Federation of Gynaecology and Obstetrics (FIGO)/AJCC Stage IB-IIIC1) that have given consent for radical radiotherapy. Induction chemotherapy prior to radiotherapy, or concurrent chemoradiotherapy acceptable.
   OR
   c. Histologically confirmed primary prostate cancer (AJCC Stage I-IIIC) that have given consent for radical radiotherapy.
2. Provide signed informed consent to participate in the study.
3. Aged ≥ 18years.

PATIENT EXCLUSION CRITERIA

1. Patients requiring radiotherapy after curative surgery or surgery that is intended to remove as much of the tumour as possible.
2. Patients receiving palliative radiotherapy.

Consecutive patients who are eligible will be approached and screening data collected to establish the proportion of eligible patients that consent to participate. Participants are only eligible for the study if they have already provided consent to receive radical radiotherapy and participation in ARCHERY will not change how the patient is treated.

For peer review only

SITE EXCLUSION CRITERIA

1. Centre using exclusively cobalt-based radiotherapy for treatment.
2. Centres not using IMRT or VMAT based radiotherapy planning for head and neck, cervical or prostate cancer.
3. Centres not using standardised international guidelines/protocols for target volume contouring and dosimetric radiation constraints

STUDY CONDUCT

The AI based RT planning tool (the RPA) will be applied to the CT scan acquired as part of a participants planned RT treatment process.

The RPA will be used to automate:

1. CTV and OAR contouring
2. Treatment planning

Randomisation is not required as the initial CT scan of the participant, which is used as the template for contouring and planning, will be used for both the standard and automated approaches. Patients will be treated on the standard plan and will not be affected by the study. Local centres will remain blinded to the results of the automated plans.

CTVs AND OAR CONTOURING

Contours will be applied manually to the GTV (for head and neck and cervical cancer) by the treating clinician.

This CT scan with the contoured GTV will be used for the manual and the automated study pathway (Figure 1) to create Plans 1 and 2. The contouring of CTVs and OARs will be performed by the clinician (blue boxes in Figure 1) or the automated by AI using the RPA (orange boxes in Figure 1).

For the prostate cancer pathway there will be no GTV to be outlined but a CTV (prostate gland +/- seminal vesicles) as per current international guidance which will be outlined by the clinician or the RPA.

TREATMENT PLANNING

Two plans will be produced for evaluation. Plan 1: Manual contours will undergo planning using standard clinical protocols by a dosimetrist or physicist at the local site, and this will be used to treat the patient (blue box in Figure 1). Plan 2: The automated contours will undergo auto-planning and this will undergo clinical evaluation (orange box in Figure 1).

For peer review only

QUALITY ASSESSMENT PROTOCOL

The final radiotherapy plans will be exported as DICOM® (Digital Imaging and Communication in Medicine) files, to the UK RT Trials Quality Assurance (RTTQA) group who will coordinate the assessment of contouring (CTVs and OARs) and planning (dose distribution) produced by the RPA. An international panel all with greater than 10 years of experience as a Radiation Oncologist will undertake the evaluation of the treatment contours using a protocol based on international standards for target volume definition and dosimetry.

PROCEDURES FOR ASSESSING EFFICACY

The primary outcome measure is overall treatment plan acceptability, a composite outcome including both the assessment of the delineated CTVs and OARs with reference to internationally accepted contouring guidelines and the adherence to pre-defined dosimetric constraints (Table 1). We will not collect patient outcomes following treatment as important clinical differences in terms of toxicity and tumour control are not expected if these validated criteria are met and patients will not be treated on the automated plans. In addition, variation in the accuracy of patient set up across the centres for treatment may result in important differences in patient outcomes unrelated to the quality of the manual or automated contours (21, 22).

The automated plan (Plan 2) will be analysed against the expected plan acceptability rate from the literature which estimates that 95% of RT plans will be acceptable (scores 3 and 4 according to study scoring criteria) for treatment without the requirement for major edits (scores of 1 or 2)(7, 23). Major edits (as defined using the Global Quality Assurance of Radiation Therapy Clinical Trials criteria), are edits that if not undertaken would affect the likelihood of cure, loco-regional control and could result in additional toxicity (24).

The assessment of the contours and dosimetry from the automated plans will be coordinated by the RTTQA group and use international standards and procedures for radiotherapy planning quality assurance which have been agreed by the Global Quality Assurance of Radiation Therapy Clinical Trials Harmonisation Group (GHG) https://rtqaharmonization.org

The RTTQA group will develop procedures and guidelines for OAR and CTV contour assessment in line with international standards for normal tissue and nodal CTVs for cervical, head and neck, and prostate cancer (25-30) that will be used by the peer reviewers in their assessments.

A scoring framework developed with reference to GHG protocol variation definitions, will be provided for assessment of contouring with examples given of contours considered

For peer review only

clinically not acceptable. The Likert scale below (with potential scope for minor changes) will be used for the grading of contouring:

- Score 4 - ACCEPTABLE – PER PROTOCOL: No edits required. The current contours are clinically acceptable and could be used for treatment without change.
- Score 3 - ACCEPTABLE VARIATION: Minor edits, not expected to have a major clinical impact on treatment outcome. Edits to the current contours should be considered.
- Score 2 - UNACCEPTABLE VARIATION: Major edits, may affect the likelihood of cure, loco-regional control and potentially cause additional toxicity. The current contours are clinically unacceptable.
- Score 1 - UNACCEPTABLE VARIATION WITH TREATMENT MODIFICATION: Major edits, however these variations could not have been reasonably avoided due to clinical necessity as perceived by the treating physician.

Two reviewers will independently review each set of contours. If there is any discordance between the two reviewers i.e., an unacceptable score (1 or 2) by one reviewer and acceptable score (3 or 4) by another, a third reviewer will be consulted, and acceptability will be defined based on a consensus majority rule (2:1).

The dosimetric assessment of the plans will be automated using the auto-extracted Dose Volume Histogram (DVH) constraints. The framework for assessment will be defined for each tumour cohort with reference to GHG criteria (31). If any of the DVH constraints are not met the plan would be assessed independently by two peer reviewers to evaluate whether there is any mitigation for the dosimetric constraints not being met (e.g., extensive tumour), otherwise the plan will be graded as clinically unacceptable.

Dosimetric constraints will be based on contemporary radiotherapy IMRT RCT protocols for cervical, head and neck, and prostate cancer (27, 32, 33) but will also require an understanding of differences in protocols used across different regions. The Likert scale below will be used for grading of DVHs:

- Score 4 - ACCEPTABLE – PER PROTOCOL: The radiation therapy treatment was planned according to protocol specifications. *Mandatory and optimal volume planning constraints are met.*
- Score 3 - ACCEPTABLE VARIATION: The radiation therapy treatment was not planned according to all the protocol specifications, but not expected to have a major clinical impact on treatment outcome. *Mandatory volume planning constraints are met but not all the optimal volume planning constraints are achieved.*

For peer review only

- Score 2 - UNACCEPTABLE VARIATION: The radiation therapy treatment was not planned according to protocol specifications and may affect the likelihood of cure, loco-regional control and potentially cause additional toxicity.
- Score 1 - UNACCEPTABLE VARIATION WITH TREATMENT MODIFICATION: The radiation therapy treatment was not planned according to protocol specifications and may affect the likelihood of cure, loco-regional control and potentially cause additional toxicity. However, these variations could not have been reasonably avoided due to clinical necessity as perceived by the treating physician.

For peer review only

Table 1: Assessment procedures for each treatment plan based on a composite score

**Contouring**	**Dosimetry**	**Overall Assessment**
Score 3 or 4	Score 3 or 4	Acceptable
Score 3 or 4	Score 1 or 2	Unacceptable
Score 1 or 2	Score 3 or 4	Unacceptable
Score 1 or 2	Score 1 or 2	Unacceptable

QUANTIFYING AND MODELLING THE TIME COST SAVINGS FROM USING THE RPA BY USING THE ESTRO APPROVED TIME-DRIVE ACTIVITY-BASED COSTING MODEL

To determine the time savings from the automated approaches compared to the manual approach, time-and-motion studies will be undertaken to populate the ESTRO time-driven activity-based costing model (TD-ABC)(6). This is considered the most contemporary and robust mechanism for radiation therapy costing and will be used to estimate the cost of delivering RT treatment for each tumour type in each centre, in addition to computing the actual personnel and equipment resource utilisation (6).

The model requires time estimates for all RT processes within the typical RT care pathway including time for contouring anatomy including edits, plan production, peer review, QA checks, plan acceptance and treatment delivery (34). All times will be stratified according to the specialist personnel involved. and undertaken for each of the two study planning pathways outlined in Figure 1. i.e., the 1. standard manual pathway 2. fully automated pathway.

Allied to this will be the collection of detailed information at the centre level of the available resources and their associated costs. This includes:

- Indirect resources: resources (and their costs) that cannot be directly assigned to a single treatment, i.e., personnel, equipment and related infrastructure and maintenance; and overheads.
- Direct material costs related to a particular treatment course (masks, fiducials, contrast material)
- Information on the number of RT courses delivered in the centre (by tumour type, treatment intent, fractionation, complexity)

The outputs from the workflow timings study and data collection on the resources available and courses delivered at each individual centre will be key inputs into the model to estimate differences in costs and resource needs for cervical cancer, head and neck cancer and prostate cancer treatment courses using the two approaches (fully automated versus standard).

Using this information, we will be able to assess the impact on resource needs and associated costs (in particular for specialist personnel e.g. ROs from scaling up automation to other cancer types, and undertake cost-minimisation and budget impact analyses (35). The costing and cost-saving calculations will be performed at the level of each country and centre , The budget impact analysis will inform the affordability of the intervention to the health system, in terms of the feasibility of expanding the number of patients that can be treated with RT(36).

SAMPLE SIZE

The sample size for each tumour population has been calculated based on an overall 95% plan acceptability rate, with a confidence interval which is no wider than +/- 5% in absolute terms around this figure This rate is considered necessary to support implementation of the software as the clinical community would be unlikely to accept a lower plan acceptability rate of, for example 80%.

To achieve at least 90% power that the lower confidence limit for the acceptability plan will be greater than 90%, assuming alpha=0.05 (or one-sided 0.025) and a binomial distribution, the required sample size for is approximately 330 for each tumour population (37).

STATISTICAL ANALYSIS PLAN

We will fit a logistic regression model to the binary outcome of plan acceptability adjusted by centre with cluster robust standard errors. We calculate the overall marginal proportion estimate balanced for centre, to calculate the robust confidence interval that maintains the family-wise error rate (FWER) at 0.05. An additional analysis will investigate the inter-rater agreement for the plan. For each cancer this will be assessed

For peer review only

by calculating the kappa statistic between the first 2 expert raters assessing plans, so excluding the conditional 3rd rating.

COMPLETION OF PARTICIPANT FOLLOW UP

Participants have no further follow up after their radiotherapy CT planning scan. Clinical management is as per the centre's standard of care.

PATIENT AND PUBLIC INVOLVEMENT

Patient partners present at the workshops have directly informed the research question, and cancer types chosen. The need for inclusion of AI and this study across LMIC partners has been defined through three international global RT workshops between 2017 and 2019, attended by the Chief Investigator (AA) at CERN (The European Organization for Nuclear Research), and in the UK and Botswana. As well as patient partners, attendees included clinical, academic, and policy stakeholders from LMICs, including Ghana, Botswana, South Africa, Nigeria, Zambia, and Jordan

With respect to study design, patient partners have highlighted that patient follow up in LMICs can be challenging, with very low rates of follow up following treatment. This has therefore informed the primary outcome measure of "radiotherapy plan acceptability" as a measure of treatment quality without the necessity for ongoing follow up. Patients will be treated using the current standard of care to avoid any delays, with the appraisal of automated plans occurring offline but using the RT CT planning scan that is acquired as part of the standard treatment process

Patients will be involved in the dissemination strategy. The patient-involvement coordinator (JT), will work with community engagement and involvement partners in each of the participating countries to develop a programme for dissemination of results.

Ethics and dissemination

The protocol has received ethical approval from UCL Research Ethics Committee and each of the six recruiting sites' Institutional Review Board (Institutional Ethics Committee, Tata Memorial Hospital; Institutional Ethics Committee, Tata Medical Centre; University of Malaya Medical Research Ethics Committee; King Hussein Cancer Center Institutional Review Board; Stellenbosch University Health Research Ethics Committee; University of Cape Town Health Research Ethics Committee). International sites will comply with the principles of GCP as laid down by the ICH topic E6 (R2) and other applicable national regulations.

Although there will be no clinical impact of the intervention or risk to the patient as they will continue to be treated according to the standard treatment pathway and not receive any additional imaging intervention or treatment, protections have been put in place for

the use of pseudonymised CT scans and treatment planning details stored as DICOM® files. Patients will be enrolled with written informed consent.

DISSEMINATION

If the study objectives are met, the AI based software will be offered as a not-for-profit web service to public sector state hospitals in LMICs. We anticipate this will support rapid adoption into daily practice and facilitate expansion of high-quality radiotherapy in these countries. This in turn will improve access, and affordability of this critical treatment option and therefore also improve survival and the quality of life of patients with cancer globally.

Authors' contributions

AA and LC conceived the original study idea. The research design was developed in conjunction with methodological experts at UCL – MP and RL; Ghent University – YL and BV; the Radiotherapy trials quality assurance group – PH, MK, EM; the study site clinical lead investigators (SL, IM, RM, JP, HS, IM) and patient and public involvement lead JT. The protocol was then further developed including study conduct, data management statistical evaluation with additional contributions from UCL – CM, MN, CR, IJ.

Competing interests

All authors: This work was supported by National Institutes of Health (NIH) National Cancer Institute (NCI) (Award No. 1U01CA269143-01) and the Rising Tide Foundation (Award No. CCR-21-300) will be providing funding for the ARCHERY study. Additional support will be provided by Medical Research Council, via UK Research and Innovation (UKRI) (core funding - MC_UU_00004/01 - Programme 1).
Ajay Aggarwal: Advancer Fellowship from National Institute for Health Research (NIHR300599)
Yolande Lievens: ESTRO University of Gent Chair on Value-Based Radiation Oncology - payments to University of Gent and unpaid member of scientific committee of ESTRO
Hannah Simonds: Contract between Stellenbosch University and MDAnderson Cancer Centre related to a different study in auto-planning radiotherapy - payment to Stellenbosch University and unpaid clinical advisory group for an auto-planning project.

Funding statement: 'This work was supported by National Institutes of Health (NIH) National Cancer Institute (NCI) (Award No. 1U01CA269143-01) and the Rising Tide Foundation (Award No. CCR-21-300) will be providing funding for the ARCHERY study. Additional support will be provided by Medical Research Council, via UK Research and Innovation (UKRI) (core funding - MC_UU_00004/01 - Programme 1)

REFERENCES

1. Atun R, Jaffray DA, Barton MB, Bray F, Baumann M, Vikram B, et al. Expanding global access to radiotherapy. The Lancet Oncology. 2015;16(10):1153-86.
2. Delaney GP, Barton MB. Evidence-based estimates of the demand for radiotherapy. Clin Oncol (R Coll Radiol). 2015;27(2):70-6.
3. Zubizarreta EH, Fidarova E, Healy B, Rosenblatt E. Need for radiotherapy in low and middle income countries - the silent crisis continues. Clin Oncol (R Coll Radiol). 2015;27(2):107-14.
4. Anakwenze CP, Ntekim A, Trock B, Uwadiae IB, Page BR. Barriers to radiotherapy access at the University College Hospital in Ibadan, Nigeria. Clin Transl Radiat Oncol. 2017;5:1-5.
5. Berumen AV. Access to medical devices for Universal Health Coverage and achieve of SDGs. World Health Organisation, Department EMaHP; 2019.
6. Defourny N, Perrier L, Borras JM, Coffey M, Corral J, Hoozee S, et al. National costs and resource requirements of external beam radiotherapy: A time-driven activity-based costing model from the ESTRO-HERO project. Radiotherapy and oncology : journal of the European Society for Therapeutic Radiology and Oncology. 2019;138:187-94.
7. Cox S, Cleves A, Clementel E, Miles E, Staffurth J, Gwynne S. Impact of deviations in target volume delineation - Time for a new RTQA approach? Radiotherapy and oncology : journal of the European Society for Therapeutic Radiology and Oncology. 2019;137:1-8.
8. Rodin D, Tawk B, Mohamad O, Grover S, Moraes FY, Yap ML, et al. Hypofractionated radiotherapy in the real-world setting: An international ESTRO-GIRO survey. Radiotherapy and oncology : journal of the European Society for Therapeutic Radiology and Oncology. 2021;157:32-9.
9. WHO. WHO guideline: recommendations on digital interventions for health system strengthening. 2019.
10. Rhee DJ, Cardenas CE, Elhalawani H, McCarroll R, Zhang L, Yang J, et al. Automatic detection of contouring errors using convolutional neural networks. Medical Physics. 2019;46(11):5086-97.
11. Cardenas CE, Anderson BM, Aristophanous M, Yang J, Rhee DJ, McCarroll RE, et al. Auto-delineation of oropharyngeal clinical target volumes using 3D convolutional neural networks. Phys Med Biol. 2018;63(21):215026.
12. Kisling K, Zhang L, Simonds H, Fakie N, Yang J, McCarroll R, et al. Fully Automatic Treatment Planning for External-Beam Radiation Therapy of Locally Advanced Cervical Cancer: A Tool for Low-Resource Clinics. J Glob Oncol. 2019(5):1-9.
13. McCarroll RE, Beadle BM, Balter PA, Burger H, Cardenas CE, Dalvie S, et al. Retrospective Validation and Clinical Implementation of Automated Contouring of Organs at Risk in the Head and Neck: A Step Toward Automated Radiation Treatment Planning for Low- and Middle-Income Countries. J Glob Oncol. 2018(4):1-11.

For peer review only

14. Court LE, Kisling K, McCarroll R, Zhang L, Yang J, Simonds H, et al. Radiation Planning Assistant - A Streamlined, Fully Automated Radiotherapy Treatment Planning System. J Vis Exp. 2018(134).
15. Kisling K, Cardenas C, Anderson BM, Zhang L, Jhingran A, Simonds H, et al. Automatic Verification of Beam Apertures for Cervical Cancer Radiation Therapy. Practical radiation oncology. 2020;10(5):e415-e24.
16. Rhee DJ, Cardenas CE, Elhalawani H, McCarroll R, Zhang L, Yang J, et al. Automatic detection of contouring errors using convolutional neural networks. Med Phys. 2019;46(11):5086-97.
17. Sung H, Ferlay J, Siegel RL, Laversanne M, Soerjomataram I, Jemal A, et al. Global Cancer Statistics 2020: GLOBOCAN Estimates of Incidence and Mortality Worldwide for 36 Cancers in 185 Countries. CA Cancer J Clin. 2021;71(3):209-49.
18. Arbyn M, Weiderpass E, Bruni L, de Sanjosé S, Saraiya M, Ferlay J, et al. Estimates of incidence and mortality of cervical cancer in 2018: a worldwide analysis. The Lancet Global Health. 2020;8(2):e191-e203.
19. Organization WH. Global strategy to accelerate the eliminaton of cervical cancer as a public health problem 2020.
20. Lievens Y, Defourny N, Coffey M, Borras JM, Dunscombe P, Slotman B, et al. Radiotherapy staffing in the European countries: final results from the ESTRO-HERO survey. Radiotherapy and oncology : journal of the European Society for Therapeutic Radiology and Oncology. 2014;112(2):178-86.
21. Yeung TK, Bortolotto K, Cosby S, Hoar M, Lederer E. Quality assurance in radiotherapy: evaluation of errors and incidents recorded over a 10 year period. Radiotherapy and oncology : journal of the European Society for Therapeutic Radiology and Oncology. 2005;74(3):283-91.
22. Shafiq J, Barton M, Noble D, Lemer C, Donaldson LJ. An international review of patient safety measures in radiotherapy practice. Radiotherapy and Oncology. 2009;92(1):15-21.
23. Brunskill K, Nguyen TK, Boldt RG, Louie AV, Warner A, Marks LB, et al. Does Peer Review of Radiation Plans Affect Clinical Care? A Systematic Review of the Literature. Int J Radiat Oncol Biol Phys. 2017;97(1):27-34.
24. Lewis PJ, Court LE, Lievens Y, Aggarwal A. Structure and Processes of Existing Practice in Radiotherapy Peer Review: A Systematic Review of the Literature. Clin Oncol (R Coll Radiol). 2021;33(4):248-60.
25. Grégoire V, Ang K, Budach W, Grau C, Hamoir M, Langendijk JA, et al. Delineation of the neck node levels for head and neck tumors: a 2013 update. DAHANCA, EORTC, HKNPCSG, NCIC CTG, NCRI, RTOG, TROG consensus guidelines. Radiotherapy and oncology : journal of the European Society for Therapeutic Radiology and Oncology. 2014;110(1):172-81.
26. Brouwer CL, Steenbakkers RJ, Bourhis J, Budach W, Grau C, Gregoire V, et al. CT-based delineation of organs at risk in the head and neck region: DAHANCA, EORTC, GORTEC, HKNPCSG, NCIC CTG, NCRI, NRG Oncology and TROG consensus guidelines. Radiotherapy and oncology : journal of the European Society for Therapeutic Radiology and Oncology. 2015;117(1):83-90.
27. Tanderup K. PR, Lindegaard J., Kiristis C, Juergenliemk-Schulz I., Leeuw A., Liederer T., Tan L. Image guided intensity modulated External beam

For peer review only

radiochemotherapy and MRI based Brachytherapy in locally advanced Cervical Cancer. EMBRACE-II 2015 [Available from: https://www.embracestudy.dk/UserUpload/PublicDocuments/EMBRACE%20II%20Protocol.pdf.
28. Salembier C, Villeirs G, De Bari B, Hoskin P, Pieters BR, Van Vulpen M, et al. ESTRO ACROP consensus guideline on CT- and MRI-based target volume delineation for primary radiation therapy of localized prostate cancer. Radiotherapy and oncology : journal of the European Society for Therapeutic Radiology and Oncology. 2018;127(1):49-61.
29. Mir R, Kelly SM, Xiao Y, Moore A, Clark CH, Clementel E, et al. Organ at risk delineation for radiation therapy clinical trials: Global Harmonization Group consensus guidelines. Radiotherapy and oncology : journal of the European Society for Therapeutic Radiology and Oncology. 2020;150:30-9.
30. NRG Oncology. Gynecologic Cancer Atlases, Templates, & Tools 2022 [Available from: https://www.nrgoncology.org/ciro-gynecologic.
31. Hansen CR, Crijns W, Hussein M, Rossi L, Gallego P, Verbakel W, et al. Radiotherapy Treatment plannINg study Guidelines (RATING): A framework for setting up and reporting on scientific treatment planning studies. Radiotherapy and oncology : journal of the European Society for Therapeutic Radiology and Oncology. 2020;153:67-78.
32. Dearnaley D, Syndikus I, Mossop H, Khoo V, Birtle A, Bloomfield D, et al. Conventional versus hypofractionated high-dose intensity-modulated radiotherapy for prostate cancer: 5-year outcomes of the randomised, non-inferiority, phase 3 CHHiP trial. The Lancet Oncology. 2016;17(8):1047-60.
33. Stelmes JJ, Vu E, Grégoire V, Simon C, Clementel E, Kazmierska J, et al. Quality assurance of radiotherapy in the ongoing EORTC 1420 "Best of" trial for early stage oropharyngeal, supraglottic and hypopharyngeal carcinoma: results of the benchmark case procedure. Radiat Oncol. 2021;16(1):81.
34. Lievens Y, Obyn C, Mertens AS, Van Halewyck D, Hulstaert F. Stereotactic body radiotherapy for lung cancer: how much does it really cost? Journal of thoracic oncology : official publication of the International Association for the Study of Lung Cancer. 2015;10(3):454-61.
35. Lievens YRD, Aggarwal A. Financial and Economic Considerations in Radiation Oncology. In: Dyk JV, editor. The Modern Technology of Radiation Oncology. 4. United States of America: Medical Physics Publishing; 2020.
36. Van Dyk J, Zubizarreta E, Lievens Y. Cost evaluation to optimise radiation therapy implementation in different income settings: A time-driven activity-based analysis. Radiotherapy and oncology : journal of the European Society for Therapeutic Radiology and Oncology. 2017;125(2):178-85.
37. Ruvuna F. Unequal Center Sizes, Sample Size, and Power in Multicenter Clinical Trials. Drug Information Journal. 2004;38(4):387-94.

For peer review only

Figure captions/footnotes:

Figure 1 caption: Study workflow

Figure 1 footnote: GTV (Gross tumour volume); CTV (Clinical target volume); OAR (Organs at risk)

For peer review only

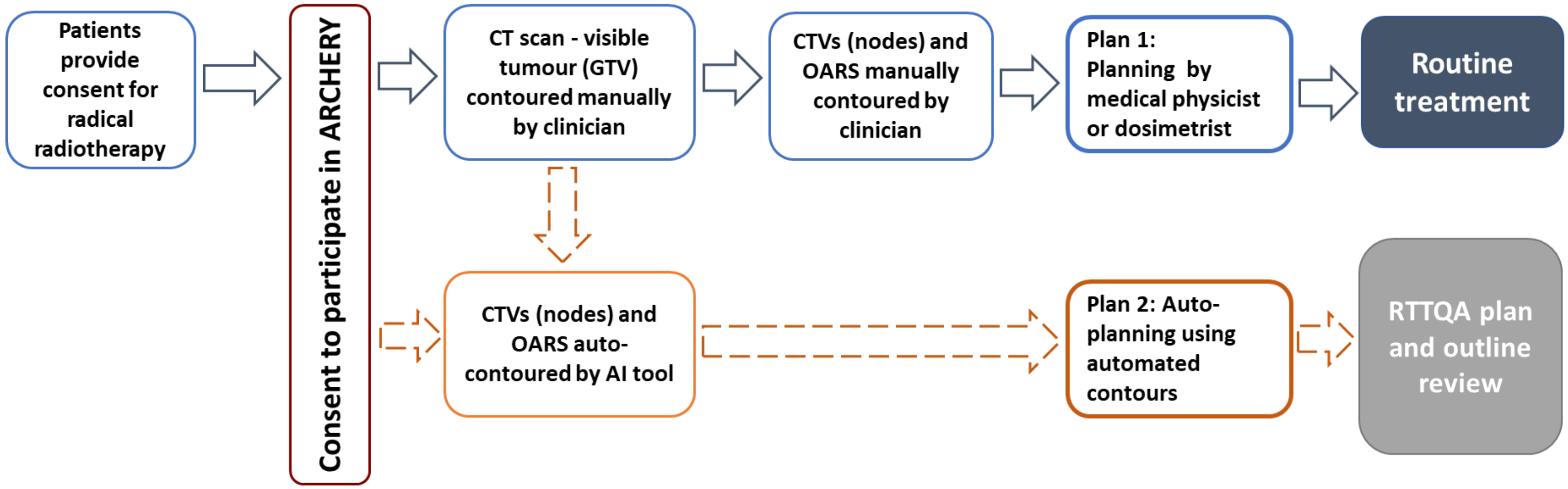
Patients provide consent for radical radiotherapy
Consent to participate in ARCHERY
CT scan - visible tumour (GTV) contoured manually by clinician
CTVs (nodes) and OARS manually contoured by clinician
Plan 1: Planning by medical physicist or dosimetrist
Routine treatment
CTVs (nodes) and OARS auto-contoured by AI tool
Plan 2: Auto-planning using automated contours
RTTQA plan and outline review

STANDARD PROTOCOL ITEMS: RECOMMENDATIONS FOR INTERVENTIONAL TRIALS

SPIRIT 2013 Checklist: Recommended items to address in a clinical trial protocol and related documents*

Section/item	Item No	Description	Page Number
**Administrative information**			
Title	1	Descriptive title identifying the study design, population, interventions, and, if applicable, trial acronym	Page IV, summary of the study
Trial registration	2a	Trial identifier and registry name. If not yet registered, name of intended registry	Page IV – ISRCTN and Clinicaltrials.gov
	2b	All items from the World Health Organization Trial Registration Data Set	Pages II - IV
Protocol version	3	Date and version identifier	Page IV, summary of study
Funding	4	Sources and types of financial, material, and other support	Page IV, summary of study
Roles and responsibilities	5a	Names, affiliations, and roles of protocol contributors	Page III, trial administration
	5b	Name and contact information for the trial sponsor	Page II under “Sponsor”
	5c	Role of study sponsor and funders, if any, in study design; collection, management, analysis, and interpretation of data; writing of the report; and the decision to submit the report for publication, including whether they will have ultimate authority over any of these activities	Page 30 – section 13.4
	5d	Composition, roles, and responsibilities of the coordinating centre, steering committee, endpoint adjudication committee, data management team, and other individuals or groups overseeing the trial, if applicable (see Item 21a for data monitoring committee)	Page 30, oversight and trial committees.
**Introduction**			

For peer review only

Background and rationale	6a	Description of research question and justification for undertaking the trial, including summary of relevant studies (published and unpublished) examining benefits and harms for each intervention	Please see Pages 1 to 5
	6b	Explanation for choice of comparators	
Objectives	7	Specific objectives or hypotheses	Page 7.
Trial design	8	Description of trial design including type of trial (eg, parallel group, crossover, factorial, single group), allocation ratio, and framework (eg, superiority, equivalence, noninferiority, exploratory)	Page IV and Pages 14 and 15

Methods: Participants, interventions, and outcomes

Study setting	9	Description of study settings (eg, community clinic, academic hospital) and list of countries where data will be collected. Reference to where list of study sites can be obtained	Please see Page 8
Eligibility criteria	10	Inclusion and exclusion criteria for participants. If applicable, eligibility criteria for study centres and individuals who will perform the interventions (eg, surgeons, psychotherapists)	Please see Page 8 and 9 for study site inclusion/exclusion criteria. Please see Page 11 regarding selection of participants.
Interventions	11a	Interventions for each group with sufficient detail to allow replication, including how and when they will be administered	Please see Page 14 and 15
	11b	Criteria for discontinuing or modifying allocated interventions for a given trial participant (eg, drug dose change in response to harms, participant request, or improving/worsening disease)	Please see Page 15
	11c	Strategies to improve adherence to intervention protocols, and any procedures for monitoring adherence (eg, drug tablet return, laboratory tests)	N/A
	11d	Relevant concomitant care and interventions that are permitted or prohibited during the trial	N/A

For peer review only - http://bmjopen.bmj.com/site/about/guidelines.xhtml

Outcomes	12	Primary, secondary, and other outcomes, including the specific measurement variable (eg, systolic blood pressure), analysis metric (eg, change from baseline, final value, time to event), method of aggregation (eg, median, proportion), and time point for each outcome. Explanation of the clinical relevance of chosen efficacy and harm outcomes is strongly recommended	Please see page 16 and 17. Please also see Page 20 and 21.
Participant timeline	13	Time schedule of enrolment, interventions (including any run-ins and washouts), assessments, and visits for participants. A schematic diagram is highly recommended (see Figure)	Please see Page 14
Sample size	14	Estimated number of participants needed to achieve study objectives and how it was determined, including clinical and statistical assumptions supporting any sample size calculations	Please see Page 21
Recruitment	15	Strategies for achieving adequate participant enrolment to reach target sample size	Recruitment not expected to be a challenge given numbers of cases treated per year in each centre and that the study does not result in any deviation from the standard treatment plan of the patient
**Methods: Assignment of interventions (for controlled trials)**			**Not Applicable**
Allocation:			
Sequence generation	16a	Method of generating the allocation sequence (eg, computer-generated random numbers), and list of any factors for stratification. To reduce predictability of a random sequence, details of any planned restriction (eg, blocking) should be provided in a separate document that is unavailable to those who enrol participants or assign interventions	N/A

For peer review only - http://bmjopen.bmj.com/site/about/guidelines.xhtml

Allocation concealment mechanism	16b	Mechanism of implementing the allocation sequence (eg, central telephone; sequentially numbered, opaque, sealed envelopes), describing any steps to conceal the sequence until interventions are assigned	N/A
Implementation	16c	Who will generate the allocation sequence, who will enrol participants, and who will assign participants to interventions	N/A
Blinding (masking)	17a	Who will be blinded after assignment to interventions (eg, trial participants, care providers, outcome assessors, data analysts), and how	N/A
	17b	If blinded, circumstances under which unblinding is permissible, and procedure for revealing a participant's allocated intervention during the trial	N/A
**Methods: Data collection, management, and analysis**			
Data collection methods	18a	Plans for assessment and collection of outcome, baseline, and other trial data, including any related processes to promote data quality (eg, duplicate measurements, training of assessors) and a description of study instruments (eg, questionnaires, laboratory tests) along with their reliability and validity, if known. Reference to where data collection forms can be found, if not in the protocol	Please see Page 19.
	18b	Plans to promote participant retention and complete follow-up, including list of any outcome data to be collected for participants who discontinue or deviate from intervention protocols	Please see Page 15. No concerns re: retention as no follow up required
Data management	19	Plans for data entry, coding, security, and storage, including any related processes to promote data quality (eg, double data entry; range checks for data values). Reference to where details of data management procedures can be found, if not in the protocol	Please see Pages 24-27 which focuses on data collection and storage of the different data types.

For peer review only - http://bmjopen.bmj.com/site/about/guidelines.xhtml

Statistical methods	20a	Statistical methods for analysing primary and secondary outcomes. Reference to where other details of the statistical analysis plan can be found, if not in the protocol	Please see Page 22 for statistical methods for analysing our primary outcome measure (plan acceptability). Our secondary outcome measure – cost savings will be done using the Time driven costing model. Please see Page 20 and 21.
	20b	Methods for any additional analyses (eg, subgroup and adjusted analyses)	N/A
	20c	Definition of analysis population relating to protocol non-adherence (eg, as randomised analysis), and any statistical methods to handle missing data (eg, multiple imputation)	N/A
**Methods: Monitoring**			
Data monitoring	21a	Composition of data monitoring committee (DMC); summary of its role and reporting structure; statement of whether it is independent from the sponsor and competing interests; and reference to where further details about its charter can be found, if not in the protocol. Alternatively, an explanation of why a DMC is not needed	Due to the nature of this particular study we will not have a DMC but a study steering committee.
	21b	Description of any interim analyses and stopping guidelines, including who will have access to these interim results and make the final decision to terminate the trial	No interim analyses planned. We will assure the quality of the data coming in.
Harms	22	Plans for collecting, assessing, reporting, and managing solicited and spontaneously reported adverse events and other unintended effects of trial interventions or trial conduct	Not applicable as there is no intervention received by the patient as part of the study
Auditing	23	Frequency and procedures for auditing trial conduct, if any, and whether the process will be independent from investigators and the sponsor	Section 7
**Ethics and dissemination**			

For peer review only - http://bmjopen.bmj.com/site/about/guidelines.xhtml

Research ethics approval	24	Plans for seeking research ethics committee/institutional review board (REC/IRB) approval	REC/IRB approvals are required for each site. Please see Page 26
Protocol amendments	25	Plans for communicating important protocol modifications (eg, changes to eligibility criteria, outcomes, analyses) to relevant parties (eg, investigators, REC/IRBs, trial participants, trial registries, journals, regulators)	Please see Page 36
Consent or assent	26a	Who will obtain informed consent or assent from potential trial participants or authorised surrogates, and how (see Item 32)	Please see Page 24 under ethical conduct. Page 8 highlights the investigators ability to create a delegate log so that appropriately qualified persons can take consent.
	26b	Additional consent provisions for collection and use of participant data and biological specimens in ancillary studies, if applicable	N/A
Confidentiality	27	How personal information about potential and enrolled participants will be collected, shared, and maintained in order to protect confidentiality before, during, and after the trial	Please see Page 25 and 26.
Declaration of interests	28	Financial and other competing interests for principal investigators for the overall trial and each study site	Please see PI qualifications and agreements P8
Access to data	29	Statement of who will have access to the final trial dataset, and disclosure of contractual agreements that limit such access for investigators	Please see Page 35.
Ancillary and post-trial care	30	Provisions, if any, for ancillary and post-trial care, and for compensation to those who suffer harm from trial participation	See section on indemnity Page 28.

For peer review

For peer review only - http://bmjopen.bmj.com/site/about/guidelines.xhtml

Dissemination policy	31a	Plans for investigators and sponsor to communicate trial results to participants, healthcare professionals, the public, and other relevant groups (eg, via publication, reporting in results databases, or other data sharing arrangements), including any publication restrictions	Please see Pages 33 and 34
	31b	Authorship eligibility guidelines and any intended use of professional writers	
	31c	Plans, if any, for granting public access to the full protocol, participant-level dataset, and statistical code	
**Appendices**			
Informed consent materials	32	Model consent form and other related documentation given to participants and authorised surrogates	Please PIS included with submission.
Biological specimens	33	Plans for collection, laboratory evaluation, and storage of biological specimens for genetic or molecular analysis in the current trial and for future use in ancillary studies, if applicable	No ancillary studies planned.

*It is strongly recommended that this checklist be read in conjunction with the SPIRIT 2013 Explanation & Elaboration for important clarification on the items. Amendments to the protocol should be tracked and dated. The SPIRIT checklist is copyrighted by the SPIRIT Group under the Creative Commons "Attribution-NonCommercial-NoDerivs 3.0 Unported" license.

For peer review only
