# Supplementary material for: ARCHERY: a prospective observational study of artificial intelligence-based radiotherapy treatment planning for cervical, head and neck and prostate cancer – study protocol
Source: BMJ Open. 2023 Dec 7;13(12):e077253. doi: 10.1136/bmjopen-2023-077253 (PMC10711912; doi:10.1136/bmjopen-2023-077253)
Supplement: Reviewer comments [file bmjopen-2023-077253.reviewer_comments.pdf]

PEER REVIEW HISTORY

BMJ Open publishes all reviews undertaken for accepted manuscripts. Reviewers are asked to complete a checklist review form (**http://bmjopen.bmj.com/site/about/resources/checklist.pdf**) and are provided with free text boxes to elaborate on their assessment. These free text comments are reproduced below.

ARTICLE DETAILS

**TITLE (PROVISIONAL)**	ARCHERY: A prospective observational study of artificial intelligence based radiotherapy treatment planning for cervical, head and neck and prostate cancer - study protocol
**AUTHORS**	Aggarwal, Ajay; Court, Laurence Edward; Hoskin, Peter; Jacques, Isabella; Kroiss, Mariana; Laskar, Sarbani; Lievens, Yolande; Mallick, Indranil; Abdul Malik, Rozita; Miles, Elizabeth; Mohamad, Issa; Murphy, Claire; Nankivell, Matthew; Parkes, Jeannette; Parmar, Mahesh; Roach, Carol; Simonds, Hannah; Torode, Julie; Vanderstraeten, Barbara; Langley, Ruth

This article was not externally reviewed at BMJ Open. Protocols that have been independently assessed prior to submission to BMJ Open are usually fast-tracked to publication on the grounds that further substantial changes will not be possible. This independent assessment will usually be external, independent review for both a substantial grant award from a non-commercial or government funder and ethics approval.
